# Modulating Th2 Cell Immunity for the Treatment of Asthma

**DOI:** 10.3389/fimmu.2021.637948

**Published:** 2021-02-10

**Authors:** Beatriz León, Andre Ballesteros-Tato

**Affiliations:** ^1^Department of Microbiology, University of Alabama at Birmingham, Birmingham, AL, United States; ^2^Division of Clinical Immunology and Rheumatology, Department of Medicine, University of Alabama at Birmingham, Birmingham, AL, United States

**Keywords:** Th2 airway inflammation, Tfh cell, asthma, cytokines, T cell

## Abstract

It is estimated that more than 339 million people worldwide suffer from asthma. The leading cause of asthma development is the breakdown of immune tolerance to inhaled allergens, prompting the immune system's aberrant activation. During the early phase, also known as the sensitization phase, allergen-specific T cells are activated and become central players in orchestrating the subsequent development of allergic asthma following secondary exposure to the same allergens. It is well-established that allergen-specific T helper 2 (Th2) cells play central roles in developing allergic asthma. As such, 80% of children and 60% of adult asthma cases are linked to an unwarranted Th2 cell response against respiratory allergens. Thus, targeting essential components of Th2-type inflammation using neutralizing antibodies against key Th2 modulators has recently become an attractive option for asthmatic patients with moderate to severe symptoms. In addition to directly targeting Th2 mediators, allergen immunotherapy, also known as desensitization, is focused on redirecting the allergen-specific T cells response from a Th2-type profile to a tolerogenic one. This review highlights the current understanding of the heterogeneity of the Th2 cell compartment, their contribution to allergen-induced airway inflammation, and the therapies targeting the Th2 cell pathway in asthma. Further, we discuss available new leads for successful targeting pulmonary Th2 cell responses for future therapeutics.

## Introduction

Asthma is a chronic lung disease characterized by breathing problems and obstructed airflow when airways swell and narrow and produce excess mucus (1). Allergic asthma is the most common form of asthma and is caused by the inhalation of allergens, which trigger the overreaction of the immune system in allergic people (1). The most common airborne allergens are pollen, fungal spores, house dust mites (HDM), and animal allergens. The characteristic pattern of inflammation in the airways of patients with allergic asthma includes the production of T helper 2 (Th2)-associated cytokines, such as interleukin- (IL-) 4, IL-13, and IL-5 by Th2 cells and type 2 innate lymphoid cells (ILC2s), the activation of mast cells, the infiltration and activation of eosinophils, and the increased production of immunoglobulin E (IgE) by B cells (2). Clinical and preclinical studies demonstrate a strong cause and effect relationship between the aberrant expansion of allergen-specific Th2 CD4^+^ T cells and the development of asthma pathogenesis, thus leading to the idea that Th2 cells play a central role in allergic asthma (1, 2).

The development of allergic asthma occurs in two phases (1, 3). Phase one requires an initial exposure to allergen or “*sensitization*” that does not necessarily cause symptoms or pathology. Phase two is characterized by pathology development following secondary or subsequent allergen exposures or “c*hallenges*.” Initial sensitization to airborne allergens occurs typically in early childhood, and it is characterized by the initial priming of allergen-specific CD4^+^ T cells with a Th2-like cytokine profile. These T cells persist after the initial priming and can be subsequently reactivated upon re-exposure with the same inhaled allergen, which caused their migration to the airways, where they locally produce Th2 cytokines. The accumulation of effector Th2 cells in the lungs ultimately stimulates the hallmark features of asthma, such as mucus hypersecretion and bronchial hyperresponsiveness (1).

Most patients with asthma achieve good disease control with the principal use of inhaled corticosteroids and bronchodilators (4, 5). However, a large proportion of patients with asthma remain poorly controlled (6). The failure of conventional therapies in these corticosteroid-resistant patients justifies looking for new approaches to treat allergic asthma. In this regard, the central role of Th2 cells in regulating airway inflammation has aroused great interest in the therapeutic potential of “*anti-Th2 approaches*.” As such, new biological asthma medications based on monoclonal antibodies against key Th2 mediators have been recently approved, and more are being under investigation (7). Furthermore, allergen immunotherapy, a long-term treatment that inhibits Th2-cell-mediated responses, decreases symptoms for many people with allergy disease (8), thereby evidencing the central pathogenic role of Th2 cells in the pathophysiology of allergy.

Here, we will review the available treatments for allergic asthma and discuss the potential immunological mechanisms underlying the clinical benefits of these therapies. Finally, recent studies provide evidence of a critical function of T follicular helper (Tfh) cells, a subset of CD4^+^ T cells that help GC B cell responses, in the allergic asthma pathogenesis. Therefore, we will discuss potential therapeutic approaches to target Tfh cells and suppress IgE responses and Th2 cell-mediated allergic inflammation in asthmatic patients.

## Pathogenic Roles of Th2 Cytokines in Allergic Asthma

**Figure 1:**
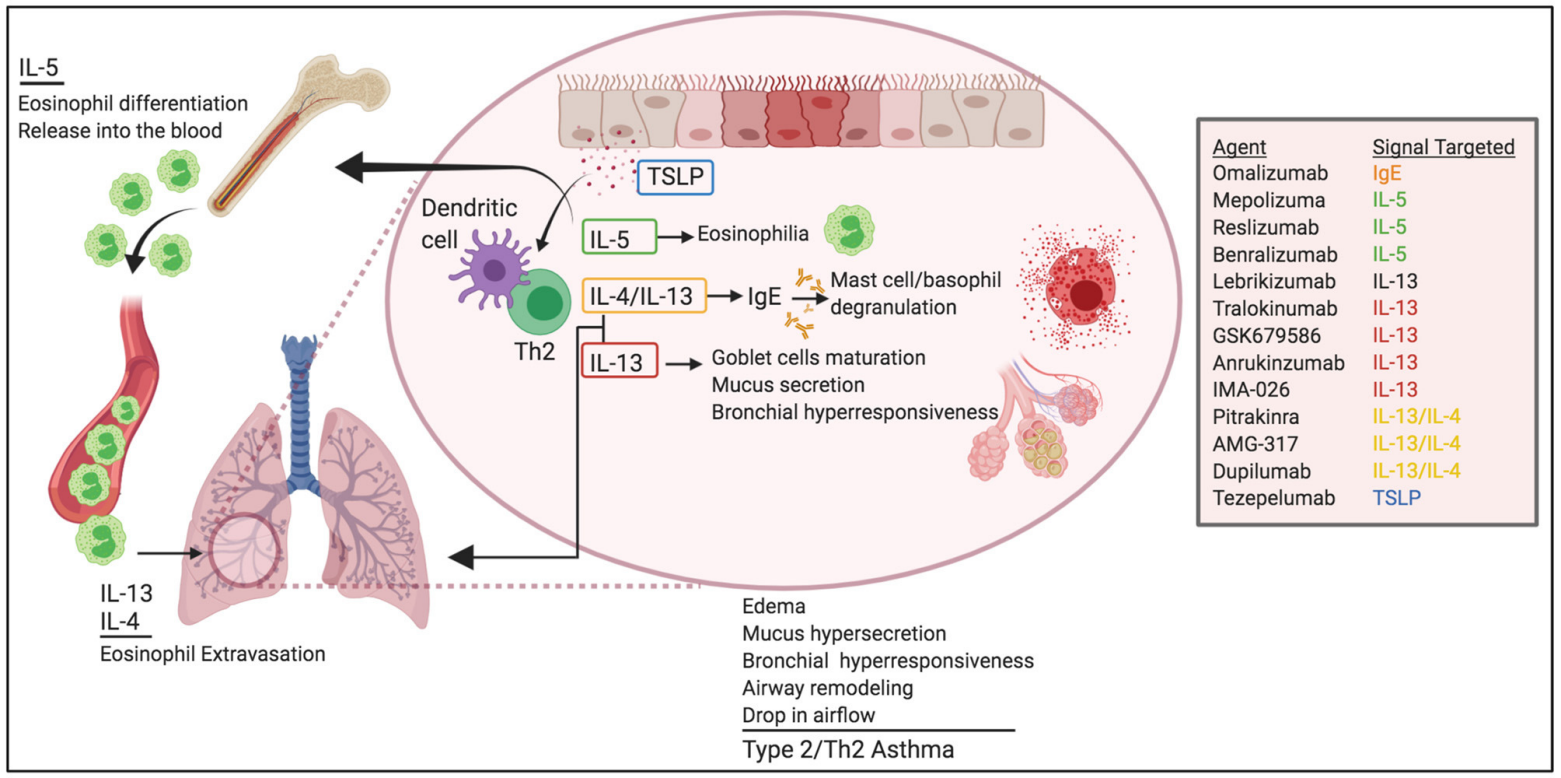
Pathogenic roles of Th2 cytokines in allergic asthma. Th2 cytokines play critical roles in asthma pathogenesis. IL-5 promotes eosinophil egress from the BM and help survival in the lungs. IL-4 and IL-13 induce eosinophil extravasation from the blood into the tissue and promote IgE class-switching. IL-13 favors globet cell maturation, mucus secretion, and airway hyperresponsiveness. TSLP helps Th2 differentiation and DC function. Altogether, these effects lead to edema, mucus hypersecretion, bronchial hyperresponsiveness and remodeling, and ultimately drop in air-flow and asthma. Novel biological therapeutics target these pathways for the treatment of asthma.

Eighty percentage of children and 60% of adults with asthma have type 2/Th2 asthma (9), which is driven by allergen-induced production of IgE and Th2 cytokines, including IL-5, IL-13, and IL-4 (Figure 1). Studies in mice, initially using OVA adjuvant and adjuvant-free sensitization protocols and most recently, using natural allergens such as HDM, cockroaches, sensitizing fungi, and protease allergens, have demarcated our knowledge on Th2 cytokines in asthma. For example, IL-4 produced by T cells drives IgE class switching (10–15) and, in conjunction with IL-13, is required to produce high-affinity IgE (16). IgE mediates mast cell and basophil degranulation by FcεRI crosslinking upon allergen recognition (17–19). Activation of FcεRI results in the immediate release of preformed granular substances (e.g., histamine, heparin, and proteases) and the production of inflammatory mediators, such as cytokines and arachidonic acid metabolites. This activation drives edema, mucus hypersecretion, and bronchial hyperresponsiveness, all accompanied by a drop in airflow in the airways. In some cases, activation of FcεRI can develop into a life-threatening systemic reaction called anaphylaxis (20).

In addition to regulating IgE production, IL-13 and IL-4 are implicated in cardinal features of asthma, such as extravasation and trafficking of eosinophils into the tissue (21–27), goblet cell maturation, mucus secretion (28), bronchial hyperresponsiveness (28, 29), and tissue remodeling (30).

IL-5 is responsible for the maturation of eosinophils in the bone marrow and their release into the blood (31). As such, IL-5 production in the airways favors the production, accumulation, and activation of eosinophils in the lung (32), and ultimately, the release of a large number of mediators capable of inducing bronchial hyperresponsiveness, mucus hypersecretion via enhanced differentiation of goblet cells (33–36) and, airway remodeling (37, 38).

Although ILC2s and other cells can also contribute to Th2 cytokines production, IL-4, IL-13, and IL-5 are principally produced by Th2 cells during ongoing chronic asthmatic responses. Given the pathogenic role of Th2 cells and Th2 cytokines, treatments for patients with type 2/Th2 asthma are directed to globally suppress Th2-mediated inflammation or to specifically target the most pathogenic effector functions of the various Th2 cytokines or the IgE response.

## Conventional Treatments that Target Th2-Type Inflammation in Asthma

Inhaled corticosteroids (ICS) are the most effective and commonly used long-term control drugs for asthma (4, 5). They locally suppress many aspects of Th2 cell-mediated inflammation, including Th2 cytokines (IL-4, IL-13, IL-5) epithelium-derived cytokines (TSLP, IL-33), chemotactic chemokines (IL-8, RANTES, MIP-1α, eotaxin, CCR2), and adhesion molecules (ICAM-1, VCAM-1) (4, 5, 39–41). Globally, ICS reduce the recruitment and maintenance of inflammatory cells into the airways of asthmatic patients, including dendritic cells, Th2 cells, eosinophils, and mast cells. Mechanistically, ICS suppress the production of chemotactic mediators, prevent the expression of adhesion molecules, and inhibit the survival of inflammatory cells in the airways (4, 5).

ICS mediate their effects through the glucocorticoid receptor (GR), an intracellular receptor and transcription factor belonging to the nuclear receptor family (39). In the absence of the ligands, GR is maintained in the cytoplasm by chaperone proteins. Upon ligand binding, GR becomes active and translocates into the nucleus to bind glucocorticoid response elements (GREs), thereby regulating the transcription of GR target genes. GR dimers and monomers can induce either transcriptional gene induction or gene repression (39, 42–44). Besides, GR can indirectly induce gene repression by GR interaction with DNA-bound transcription factors such as NF-κB and activator protein-1 (AP-1), resulting in the repression of the respective inflammatory signaling cascades (39, 45, 46).

The wide range of anti-inflammatory effects of ICS probably accounts for their clinical effectiveness in managing type 2/Th2-asthma. Regular treatment with ICS (alone or in combination with bronchodilators, such as long-acting β2 agonists (LABAs) or Theophylline) can effectively control chronic symptoms and prevent asthma attacks in most of the patients (4, 5). However, in patients with moderate to severe asthma, ICS are less effective. Hence, unacceptably high doses of ICS or even oral corticosteroids may be required to achieve optimal control.

Several mechanisms can contribute to the reduced responsiveness to ICS in moderate/severe asthma [for a review, see (6)]. For example, cytokines such as IL-1, TNFα, nitric oxide (NO), IL-13, and IL-4, which are overexpressed in the airways of patients with corticosteroid-resistant asthma, have been shown to reduce GR nuclear translocation and function. Ultimately, people with severe asthma are refractory to ICS treatment and experience poor symptom control. Additionally, these patients can have frequent asthma exacerbations, in which symptoms flare-up and get progressively worse, leading to respiratory failure. Therefore, new treatments have emerged for selected patients with moderate to severe type 2/Th2 asthma disease and inadequate responsiveness to ICS. These new therapeutic avenues are aimed to target cytokines and mediators that promote type 2/Th2 immunity.

### Biologic Drugs that Target Th2-Type Inflammation in Asthma

The clinical characteristics of moderate/severe asthma disease are frequent asthma exacerbations (>2 episodes in 12 months period), high blood counts of eosinophils and sputum eosinophils, and poor response to high dosage ICS/ LABAs (47). These uncontrolled symptoms place patients at high risk for hospitalization and reduced health-related quality of life. Therefore, additional therapeutics are needed for those patients whose severe asthma does not respond well to conventional anti-inflammatory treatment. Several biologics designed to target specific mediators of Th2-type cell immunity have been proved to be effective as add-on treatments for severe asthma patients (Figure 1).

#### Anti-IgE Therapy in Severe Asthma: Omalizumab

Omalizumab is a humanized IgG1 monoclonal antibody that specifically binds to free IgE and prevents it from binding to the high-affinity IgE receptor (FcεRI) on basophils and mast cells. As Omalizumab depletes free IgE, it further promotes FcεRI down-regulation in basophils and mast cells, rendering those cells much less sensitive to stimulation by allergens and consequent degranulation (48–50).

Omalizumab is given by subcutaneous injection every 2–4 weeks. It is FDA-approved to treat moderate-to-severe asthma in patients over 6 years of age that have sensitivity to perennial aeroallergens (e.g., dust mites, pet dander, cockroach debris). The appropriate doses are determined on a combination of age, IgE levels, and body weight. In clinical trials and observational studies with moderate to severe persistent asthma patients, Omalizumab has been shown to decrease the incidence of asthma exacerbations and emergency visits by 38 and 47%, respectively, compared with controls (50).

Some potential adverse reactions have been described related to long-term effects on cardiovascular and cerebrovascular events. However, the available studies limit the ability to quantify the magnitude of the risk (50, 51). Omalizumab has also been associated with life-threatening systemic allergic anaphylactic reactions; thus, anyone who gets an injection of this drug should be monitored closely by health professionals (50).

#### Anti-IL-5 Therapy in Severe Asthma: Mepolizumab, Reslizumab, and Benralizumab

Three different biologic drugs targeting IL-5 signaling are available, and FDA-approved. All three treatments have been consistently shown to reduce blood eosinophil counts and sputum eosinophils (47, 52, 53). ***Mepolizumab***is a humanized IgG1 monoclonal antibody that recognizes and blocks IL-5 and prevents its binding to IL-5 receptor alpha subunit (IL-5Rα or CD125) on the surface of eosinophils. ***Reslizumab***is a humanized IgG4 monoclonal antibody against IL-5 that likewise prevents IL-5 function in eosinophils. Finally, ***Benralizumab***also targets IL-5-mediated effects on eosinophils, but in this case, it is via a humanized IgG1 monoclonal antibody directed against IL-5Rα/CD125. Besides, blocking IL-5/IL-5R signaling, Benralizumab induces antibody-mediated eosinophil depletion (54) and as such, very rapid eosinophil reduction in sputum, bone marrow and blood (53).

Targeting the biological activity of IL-5 with Mepolizumab, Reslizumab and Benralizumab reduces asthma exacerbations and life-threatening emergencies in corticosteroid-resistant severe eosinophilic asthma, as well as help minimize corticosteroid use (55–69). However, no consistent benefits have been shown to improve daily asthma symptoms and quality-of-life, pertaining to the use of short-acting bronchodilators, nigh awakenings, or the limitation of activities (55–57, 62, 66, 67, 70). Likewise, targeting IL-5 does not improve asthma control in patients with mild-to-moderate eosinophilia (59, 71–73). Hence, while these findings highlight the importance of eosinophils in the pathogenesis of asthma exacerbations, they also suggest that the inflammatory cues driving the day-to-day symptoms are different from the eosinophil-driven mechanisms responsible for asthma attacks. Therefore, the primary target population for these medications is limited, at best, to patients with moderate-to-severe eosinophilia and a history of frequent exacerbations.

The three current FDA-approved anti-IL-5 therapies have different administration routes and schedules. Mepolizumab is given as an at-home monthly subcutaneous injection and approved as an add-on treatment for patients 6 and older. Reslizumab is a personalized, weight-based intravenous injection given every 4 weeks and approved for use with other asthma medicines in patients aged 18 and older. Due to the risk of an anaphylactic reaction, patients should be observed after drug administration. Benralizumab is an add-on maintenance treatment for patients 12 and older and is administered once every 4–8 weeks by subcutaneous injection. A healthcare professional should oversee Benralizumab administration due to the risk of anaphylaxis.

#### Anti-IL-13/4 Therapy in Severe Asthma

Due to the central role of IL-13 and IL-4 in controlling critical aspects of asthma pathophysiology, several biologic drugs have been designed to block either IL-13 alone or IL-13 and IL-4 simultaneously. IL-13 signals primarily through the Type-2 IL-4 receptor, which is composed of two chains, IL-13Rα and IL-4Rα IL-4 can signal through both, the Type-2 IL-4 receptor and the Type 1 IL-4 receptor (consisting of IL-4Rα and common γ chain).

IL-13 alone blocking drugs include monoclonal antibodies against IL-13 such as ***Lebrikizumab***(humanized IgG4), **Tralokinumab** (human IgG4), ***GSK679586***(humanized IgG1), **Anrukinzumab** (IMA-638; humanized IgG1) and **IMA-026 (**humanized IgG1). Simultaneous targeting of IL-4 and IL-13 signaling has been achieved by using a human IL-4 mutein that competes with IL-13 and IL-4 for binding to the IL-4Rα (**Pitrakinra)**, and by using monoclonal antibodies against IL-4Rα (**AMG-317**, human IgG2 and **Dupilumab**, human IgG4).

IL-13 blocking agents show evidence of IL-13 pathway inhibition, such as a reduction in biomarkers of Th2/eosinophilic airway inflammation and serum IgE concentration. However, they do not consistently show clinically meaningful improvements in asthma control, pulmonary function, or exacerbations in severe asthma patients (74–83), most likely due to the inability of IL-13 blocking agents to reduce airway eosinophilia in humans significantly (79, 83). Collectively, these results do not support the use of Lebrikizumab, Tralokinumab, GSK679586, Anrukinzumab, and IMA-026 for the treatment of severe asthma.

The biologic activities of IL-14 and IL-13 significantly overlap. Thus, the relatively low efficacy of IL-13 blocking agents is likely due to the capacity of IL-4 and other inflammatory mediators to compensate for the lack of IL-13. Therefore, dual targeting of IL-13 and IL-4 has been suggested as a superior approach to reduce airway eosinophilia and other activities associated with airway inflammation, fibrosis, and mucus production (84). In agreement with this idea, local (inhaled) treatment with Pitrakinra, an IL-4 mutein that simultaneously blocks IL-13 and IL-4 signaling, has shown clinical efficacy in reducing asthma symptoms in a phase 2a study in patients with mild asthma (85). In a later larger study, inhaled Pitrakinra showed significant clinical efficacy in reducing the rate of exacerbations in patients with moderate-to-severe eosinophilic asthma (86). Despite these promising data, further development of Pitrakinra for asthma has ceased.

Additionally, two monoclonal antibodies to IL-4Rα have been developed for the dual inhibition of IL-4/13 signaling (AMG-317 and Dupilumab). AMG-317 displayed relatively poor pharmacokinetics and did not demonstrate clinical efficacy in a clinical trial with moderate-to-severe asthma patients (87). Dupilumab, however, has shown clinical improvements in reducing asthma exacerbations and asthma symptoms and control, as well as lung function in patients with persistent, moderate-to-severe asthma and elevated eosinophil levels (88–91). Besides, Dupilumab appears to have a more significant effect in improving bronchial hyperreactivity than inhibitors of IL-5 and significantly reduce levels of Th2-associated inflammatory indicators, including markers of eosinophilic airway inflammation and IgE levels (88, 89). IL-4 and IL-13 are essential factors promoting Th2 cell differentiation and class switching into IgE in B cells (1), but at the same time, precluding the differentiation of regulatory T cells (Tregs) (92–95). Therefore, the blockade of the actions of IL-4 and IL-13 with Dupilumab could potentially alter the course of adaptive immune responses to allergens and thus cause a long-term tolerogenic effect. If this is confirmed, Dupilumab could be considered not only a Th2-targeted therapy but an immunomodulatory therapy as well.

Up until now, Dupilumab is the only FDA-approved dual inhibitor of IL-4 and IL-13. It is currently used as an add-on maintenance treatment in patients with moderate-to-severe asthma aged 12 years and older with an eosinophilic phenotype or oral corticosteroid-dependent asthma. It is also approved for inadequately controlled chronic rhinosinusitis with nasal polyposis and atopic dermatitis (96–98). The drug is administered once every 2 weeks by subcutaneous injection and is administered at home or in office.

Interestingly, though Dupilumab decreases bronchial hyperreactivity, serum IgE, and pulmonary eosinophilia, eosinophil counts in blood are elevated (88, 89). This observation is not entirely surprising since, rather than inhibiting eosinophil differentiation, the likely mechanism by which IL-4/IL-13 blockade prevents airway eosinophilia is by precluding eosinophils recruitment from the blood into the tissues (21–27). Notably, IL-5 stimulates eosinophil development, maturation, and egress from bone marrow (31). As a result, anti-IL-5-based therapies significantly reduce eosinophil numbers in both blood and sputum (47, 52, 53). Therefore, combined blockade of multiple Th2-associated cytokines (IL-13, IL-4, and IL-5) may be a better approach to overcome cytokine redundancy and gain full control of asthma symptoms, including exacerbations, lung function, and quality of life, by simultaneous optimization of airway hyper-reactivity, eosinophil, and IgE targeting (99).

#### Promising New Therapy in Severe Asthma Targeting the Epithelial-Cytokine TSLP: Tezepelumab

The epithelial cell-derived cytokine thymic stromal lymphopoietin (TSLP) has been described as a central regulator of Th2 cell-mediated inflammation in asthma (100–104). Several studies have shown that the airways of asthmatic patients have increased TSLP expression, which correlates with higher Th2 cell response and disease severity (100–103, 105). *In vitro* approaches and *in vivo* animal models have demonstrated that TSLP is released by the barrier epithelium in response to external insults, particularly to allergens with proteolytic activity, such as HDM, cockroaches, ragweed, *Alternaria, Aspergillus*, and papain (106–113). Additional preclinical studies demonstrate that the lack of TSLP signaling results in reduced Th2 cell-mediated airway inflammation (106, 114, 115). On the contrary, TSLP overexpression leads to spontaneous Th2 cell-mediated airway inflammation and an asthma phenotype (115, 116). Mechanistically, TSLP can directly stimulate naïve CD4^+^ T cells to commit to the Th2 cell lineage (106, 114, 117) and directly stimulate dendritic cells (103, 106, 113, 115, 118, 119) and ILC2 (106, 113, 120–122) for priming Th2 cell responses.

Based on the central role of TSLP in the initiation and maintenance of Th2-cell-mediated inflammation, including not only asthma but also atopic dermatitis and food allergy (123), a human IgG2 monoclonal antibody with the ability to neutralize TSLP (***Tezepelumab***) was developed (124) and have shown promising results in severe, uncontrolled asthma (125–127). Tezepelumab was given as an add-on therapy to patients whose asthma was uncontrolled despite the use of ICS. It was found to reduce asthma exacerbations, allergen-induced bronchoconstriction, and airway inflammation indexes, including decreased levels of blood and sputum eosinophils. These findings are being further explored in an ongoing phase 2/3 trial that will produce data by early 2021. Current trials are testing *Tezepelumab* when given subcutaneously every 4 weeks. Additionally, an inhaled anti-TSLP antibody will be studied in a 652-patient Phase II study (NCT04410523) that has yet to start recruiting.

## Allergen Immunotherapy or Allergen Desensitization

Allergen immunotherapy, also known as desensitization, is a long-term medical treatment that decreases symptoms and prevents the development of allergic asthma in patients with environmental allergies (128–131). Contrary to ICS, oral corticoids, LABAs, and biologic drugs, which require continuous utilization to keep asthma symptoms under control, allergen immunotherapy is a disease-modifying approach. In these therapies, patients are exposed to gradually increasing doses of environmental allergies to divert their pathogenic Th2 cell responses from pathogenic to tolerogenic. The treatment requires a significant commitment since it usually takes 3–5 years to achieve clinical benefits. However, it often leads to long-lasting relief of allergy symptoms and severity of asthma, with an observed efficacy duration of 7–12 years after treatment is stopped (129–135). Allergen Immunotherapy may also decrease the development of new sensitizations to other allergens in both pediatric and adult patients (8, 131).

Despite proven efficacy, the mechanisms of allergen immunotherapy remain not entirely understood. Multiple overlapping mechanisms, mediators, and cell types are likely responsible for re-directing the established Th2/IgE-dominant response and the restoration of the immune tolerance to the aeroallergens. Desensitization of FcεRI-bearing mast cells and basophils, accompanied by decreased activity for degranulation and anaphylactic reactions, is observed early after treatment. This effect could be mediated by the up-regulation of the histamine type 2 receptor, which has a suppressive effect on the activation of mast cells and basophils (136). As the therapy progresses, IgG-dependent inhibition of mast cell/basophil activation might contribute to sustaining inhibition of mast cell/basophil activity. In this regard, it has been shown that specific-IgE levels in blood progressively decrease during allergen immunotherapy. On the contrary, the titters of allergen-specific IgG4 antibodies increases over time (137–142). This change in balance is thought to be the consequence of increased IL-10 production (140), which can drive allergen-specific B cells to produce IgG4 at the expense of IgE secretion (143). Although the exact clinical consequences of these changes remain unclear, it has been suggested that IgG4 can sequester antigen, thereby limiting its availability for cross-linking of receptor-bound IgE. Alternatively, IgG4 can co-stimulate the inhibitory IgG receptor FcγRIIb, which negatively regulates FcεRI signaling and cell activation (144).

Phenotypic and functional changes in the allergen-specific T cell response have been observed in the peripheral blood and nasal mucosa of treated patients. These changes included diminished production of Th2 cytokines (IL-4, IL-13, IL-5) by allergen-specific T cells (142–148) and elevated numbers of allergen-induced Foxp3^+^CD25^+^ Tregs expressing IL-10 and TGF-beta (139, 142, 146, 149–152). Whereas, the exact mechanisms through which allergen immunotherapy drives inhibition, deletion, exhaustion, replacement, or reprogramming of T cells remain elusive, changes in the cytokine milieu could partially account for these changes. For example, allergen immunotherapy triggers IL-10 induction by multiple cell types (138, 140, 153, 154). In turn, IL-10 can control Th2 cell-mediated allergic inflammation by both direct and indirect mechanisms. On the one hand, intrinsic IL-10 signaling may limit Th2 cell responses by directly inducing Th2 cell death (155). On the other hand, IL-10 might prevent Th2 cell expansion by down-regulating antigen presentation by reducing MHCII class II expression (156, 157) or via IgG4-mediated inhibition of IgE-facilitated allergen presentation (140, 158–160). The subsequent reduction in the production of Th2 cytokines, most crucially in IL-4, could favor the differentiation of allergen-specific, IL-10-producing inducible Tregs by allowing TGF-beta-dependent up-regulation of FOXP3 in responding T cells (92–95). Thus, initiating a positive feedback loop of IL-10 signaling and Treg-mediated immunosuppression that ultimately suppresses the differentiation and function of newly formed allergen-specific Th2 cells (149, 161).

In current clinical practice in the United States, immunotherapy is delivered either subcutaneously or sublingually. Additionally, other methods of allergen delivery are being tested for improving outcome.

### Subcutaneous Immunotherapy (SCIT)

Subcutaneous immunotherapy (SCIT), also known as allergy shots involves receiving subcutaneous injections of a particular aeroallergen that has been identified to cause the allergic reaction. Allergen identification is based on the presence of IgE antibodies specific to that allergen (162). Injectable allergen extracts are available to treat allergies triggered by common airborne allergens such as pollen, mold, dust mites, and animal dander.

SCIT treatment consists of two phases: During the ***Build-up phase***, the antigen is given frequently (one to two times per week) in gradually increasing doses until achieving an effective targeted dose (that reduce disease severity from natural exposure). This phase usually lasts 3–6 months. During the ***maintenance phase***, the targeted dose of allergen is injected every 3–4 weeks for at least 3–5 years. Allergy shots are recommended for people with allergy symptoms who do not respond well to usual mediations, have significant side effects from their mediation, want to reduce the long-term use of allergy medication, or for whom allergies might become life-threatening (8). Although allergen immunotherapy is generally safe, it can have adverse reactions, including anaphylaxis (163, 164). For that reason, each injection is administered in a setting with trained professionals and equipment to treat anaphylaxis (8). Further, it is essential to identify any patient characteristics (such as severe uncontrolled asthma) that may increase the risk of a severe reaction (165).

### Sublingual Immunotherapy (SLIT)

SLIT involves administering the allergens in a tablet form under the tongue, generally on a daily basis. Sublingual tablets are intended for the treatment of allergic rhinitis and allergic asthma. This therapeutic approach is available for different species of grass pollen and dust mites. SLIT can achieve a significant clinical improvement but shows less efficacy than SCIT, which offers earlier and robust clinical effects and induces systemic changes (166–169). SLIT only provides local changes in the oral mucosa and regional lymph nodes (170, 171). The significant advantage of SLIT over SCIT is its safety profile, which allows for administering this treatment outside of the medical setting after the first dose (131, 172). Still, as for the possibility of severe allergic reactions from SLIT, an epinephrine auto-injector is usually prescribed to treat potential severe reactions at home.

### Future Approaches in Allergen Immunotherapy

Although SCIT and SLIT are efficacious in that both offer significant clinical improvements in allergic and asthma symptoms, the adherence with the current regimens is low. Most likely because of the frequency of administrations and the long duration of the therapeutic courses. Thus, there is a need for more effective allergen immunotherapy strategies, especially for patients with refractory allergic disease or those who suffer adverse drug reactions.

One of the novel approaches includes using adjuvants such as Toll-like receptor (TLR) agonists. Lipopolysaccharide (LPS), also known as endotoxin, is a major component of Gram-negative bacteria that activates the innate immune response through TLR4. Exposure to airborne allergens containing endotoxin protects against asthma by suppressing the Th2 cell differentiation program in allergen-specific T cells (173–175). In this regard, monophosphoryl Lipid A (MPL), which is a TLR4 agonist, being a derivate of Lipid A from LPS that triggers a moderate inflammatory reaction (176, 177), have been evaluated in allergen immunotherapy. Compared to conventional allergen desensitization strategies, MPL immunotherapy show lasting clinical benefits even when administered in shorter courses (178–186). These results are certainly promising and encourage further controlled studies to evaluate clinical and immunological measurements and long-term efficacy.

Outside of TLR4, other agonists targeting alternative TLRs are being investigated in the context of allergen immunotherapy, with components targeting TLR9, TLR8, and TLR7. TLR9 agonists have been shown to reduce allergic symptoms and modulate the immune response to allergens when administered as an adjuvant in allergen immunotherapy (187–189). Despite promising data, clinical trials have not yet progressed beyond initial studies. TLR7 agonists are currently being evaluated for their safety in the context of allergen immunotherapy (190–193). Future studies will determine whether those are promising adjuvants.

Finally, other routes of allergen administration have been tested. Intralymphatic immunotherapy has shown favorable results in shorten treatment duration. Hence, it might offer an alternative approach to improving allergen immunotherapy adherence and success (194). Intralymphatic immunotherapy involves the application of the allergen directly into the lymph nodes. The whole treatment consists of three ultrasound-guided injections into the inguinal lymph nodes 1 month apart. Although the clinical results are favorable, more extensive studies are needed to support long-term effectiveness.

## Future Therapeutic Targets: Tfh Cells in Asthma

Experimental mouse models of allergic asthma have been instrumental in investigating the mechanisms underlying the initiation and maintenance of allergen-specific Th2 cell responses. Using these preclinical models, it has been shown that the development of allergic Th2 cell responses is more complex than initially expected. During the initial sensitization through the intranasal (i.n.) route, lung-migratory dendritic cells traffic into the lung-draining lymph nodes to prime allergen-specific CD4^+^ T cells (3, 195). Importantly, however, this initial exposure does not typically result in the accumulation of effector allergen-specific Th2 cells in the airways (1, 3). Instead, allergen sensitization triggers a strongly biased Tfh cell response that is restricted to the lung-draining lymph nodes (1, 3, 196).

**Figure 2:**
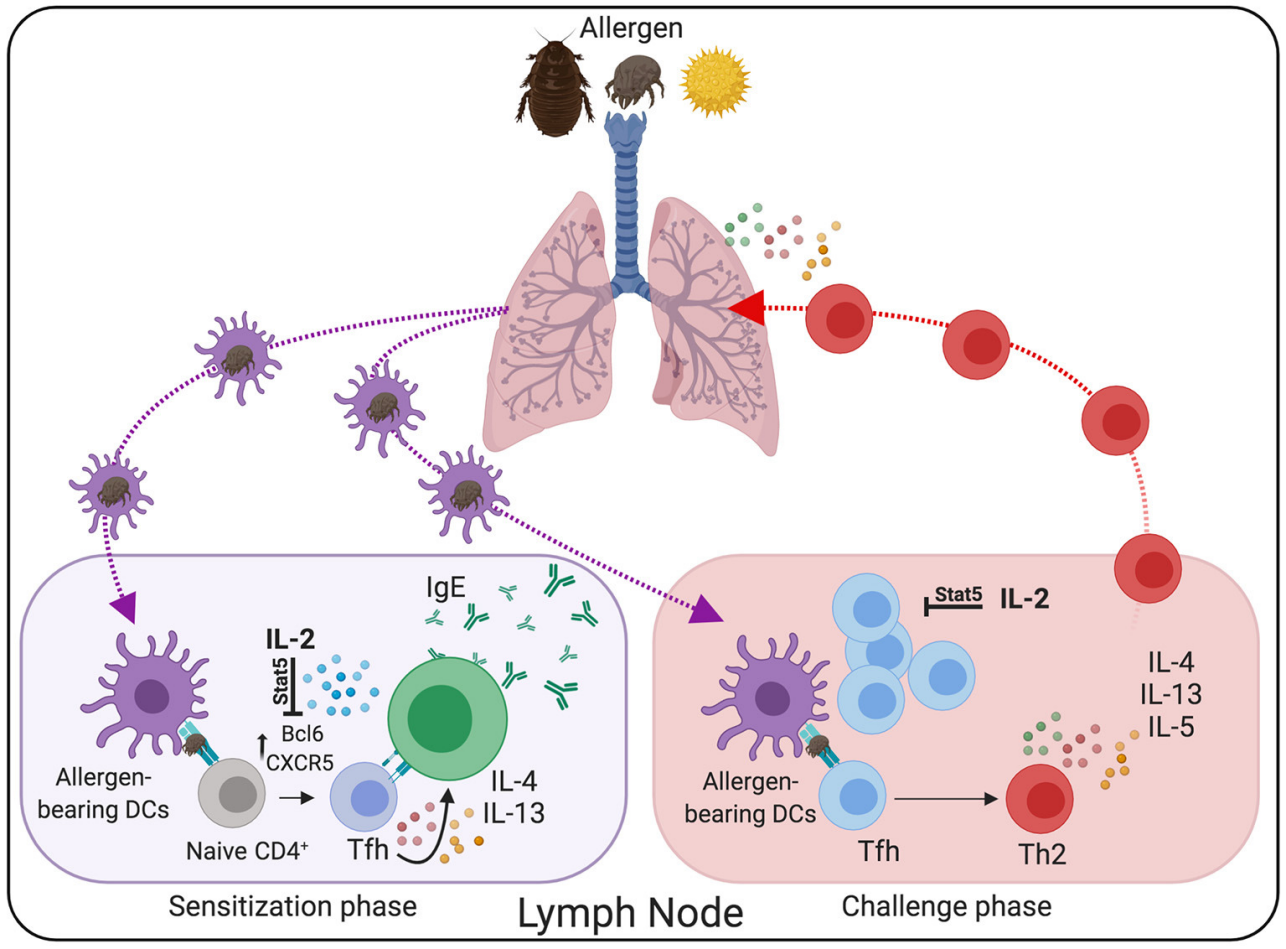
Tfh cells are critical mediators in the pathogenesis of allergic asthma. During the sensitization phase, lung-migratory DC primed allergen-specific “Type 2” Tfh cell responses in the lung-draining lymph node. Through the interaction with B cells, Type-2 Tfh cells promote IgE secretion. Following re-challenge, Tfh cells differentiate into conventional effector Th2 cells that subsequently migrate to the lung and promote allergic airway inflammation. Treatment with rIL-2 has the potential to prevent Tfh cells differentiation and maintenance, thereby reducing asthma pathogenesis.

Tfh cell development depends on the expression of the transcription factor Bcl6, which functions as a transcriptional repressor that prevents the acquisition of T effector programs, thereby facilitating Tfh cell differentiation (197–199). However, the capacity of Bcl6 to repress alternative T effector fates is not absolute. As such, whereas Tfh cells were initially characterized as IL-21-producing cells (198, 199), they are more plastic than expected and can initiate secondary differentiation programs and secrete Th1 (200–202), Th2 (3, 203), and Th17 (204) effector-like cytokines when developing in high polarizing environments. Correspondingly, work by us (3, 205), and others (10–12, 16, 206–208), show that Tfh cells can produce large amounts of Th2 cytokines, including IL-4 and IL-13, in response to allergens and helminths. Notably, while early studies considered that Th2 cells were the primary source of type 2 cytokines, it is increasingly accepted that Tfh cells, and not effector Th2 cells, are the main providers of IL-4 and IL-13 during the sensitization phase (3, 16). Furthermore, more recent data demonstrate that allergen-specific Tfh cells are critical mediators in the pathogenesis of allergic asthma (Figure 2). For example, IL-4/IL-13 producing Tfh cells are critical for the sustained production of high-affinity, allergen-specific IgE (1, 10, 16), which, as aforementioned, plays a crucial role in asthma pathogenesis. In addition, using an HDM sensitization and challenge model of asthma, we have recently found that type-2 Tfh cells survive in the lymph nodes for extended periods as memory cells and have the unique ability to give rise to effector Th2 cells upon allergen rechallenge (3). Combining fate-mapping and adoptive transfer experiments, we demonstrated that allergen-specific Tfh cells generated during the sensitization phase were the precursors of effector Th2 cells found in the lung after secondary challenge. Supporting the role of Tfh cells as progenitors of Th2 cells, depletion of Tfh cells during the sensitization phase prevented the accumulation of effector Th2 cells in the airways after challenge, thereby inhibiting asthma pathogenesis. Thus, our work establishes the lineage flexibility of Tfh cells in allergic disease and identifies these cells as a crucial long-term reservoir of Th2 cell progenitors.

All these studies collectively show a critical function of Tfh cells in allergic asthma pathogenesis, thus highlighting Tfh cells as an attractive target for the suppression of IgE responses and Th2 cell-mediated allergic inflammation. Unfortunately, there are currently no therapies to selectively target Tfh cells *in vivo*. Thus, a better understanding of the cellular and molecular mechanisms that control allergen-specific Tfh cell development and function will be critical for designing new therapeutic approaches to prevent Tfh-cell-mediated pathology in asthmatic patients. Interestingly, a large body of evidence indicates that IL-2 is a potent inhibitor of Tfh cells (3, 209–214). IL-2/STAT5 signaling prevents Tfh cell differentiation by repressing the expression of Bcl6, the master regulator of Tfh cells. As a consequence of the inhibitory effect of IL-2, Tfh cells fail to differentiate and are efficiently depleted after exogenous recombinant IL-2 treatment (3, 212, 214–217). Importantly, subcutaneous administration of low-dose recombinant human IL-2 r-IL2, (Aldesleukin/Proleukin) has potent immunosuppressive effects in patients with autoimmune disorders and can be safely administered to humans (217–220). In agreement with the role of IL-2 as an “*anti-Tfh*” agent, treatment of active Systemic Lupus Erythematosus (SLE) patients with low-dose rIL-2 resulted in reduced frequencies of Tfh cells in a recent clinical study by Jing He and colleagues (217), hence evidencing the therapeutic potential of IL-2 to prevent unwanted Tfh cell responses (Figure 2). Given the efficacy and safety of the low-dose IL-2- treatments and the putative role of Tfh cells in asthma pathogenesis, IL-2-based therapies, alone or in combination with other strategies, could represent a promising therapeutic approach to deplete allergen-specific Tfh cells and prevent allergic asthma pathogenesis.

## Author Contributions

All authors listed have made a substantial, direct and intellectual contribution to the work, and approved it for publication.

## Conflict of Interest

The authors declare that the research was conducted in the absence of any commercial or financial relationships that could be construed as a potential conflict of interest.

## References

[1] Leomicronn B. T cells in allergic asthma: key players beyond the Th2 pathway. Curr Allergy Asthma Rep. (2017) 17:43. 10.1007/s11882-017-0714-1 28555329

[2] Boonpiyathad T Sozener ZC Satitsuksanoa P Akdis CA Immunologic mechanisms in asthma. Semin Immunol. (2019) 46:101333 10.1016/j.smim.2019.101333 31703832

[3] Ballesteros-Tato A Randall TD Lund FE Spolski R Leonard WJ Leon B. T Follicular helper cell plasticity shapes pathogenic T helper 2 cell-mediated immunity to inhaled house dust mite. Immunity. (2016) 44:259–73. 10.1016/j.immuni.2015.11.017 26825674 PMC4758890

[4] Hossny E Rosario N Lee BW Singh M El-Ghoneimy D Soh JY. Le Souef, The use of inhaled corticosteroids in pediatric asthma: update. World Allergy Organ J. (2016) 9:26. 10.1186/s40413-016-0117-0 27551328 PMC4982274

[5] Barnes PJ Inhaled corticosteroids. Pharmaceuticals. (2010) 3:514–40. 10.3390/ph3030514 27713266 PMC4033967

[6] Barnes PJ. Corticosteroid resistance in patients with asthma and chronic obstructive pulmonary disease. J Allergy Clin Immunol. (2013) 131:636–45. 10.1016/j.jaci.2012.12.1564 23360759

[7] Doroudchi A Pathria M Modena BD. Asthma biologics: Comparing trial designs, patient cohorts and study results. Ann Allergy Asthma Immunol. (2020) 124:44–56. 10.1016/j.anai.2019.10.016 31655122 PMC6911637

[8] Cox L Nelson H Lockey R Calabria C Chacko T Finegold I. Allergen immunotherapy: a practice parameter third update. J Allergy Clin Immunol. (2011) 127:S1–55. 10.1016/j.jaci.2010.09.034 21122901

[9] Masuda S Fujisawa T Katsumata H Atsuta J Iguchi K. High prevalence and young onset of allergic rhinitis in children with bronchial asthma. Pediatr Allergy Immunol. (2008) 19:517–22. 10.1111/j.1399-3038.2007.00675.x 18221475

[10] Kobayashi T Iijima K Dent AL Kita H. Follicular helper T cells mediate IgE antibody response to airborne allergens. J Allergy Clin Immunol. (2017) 139:300–13.e7. 10.1016/j.jaci.2016.04.021 27325434 PMC5115999

[11] Meli AP Fontes G Leung Soo C King IL. T Follicular helper cell-derived IL-4 is required for IgE production during intestinal helminth infection. J Immunol. (2017) 199:244–52. 10.4049/jimmunol.1700141 28533444

[12] Noble Zhao J. Follicular helper T cells are responsible for IgE responses to Der p 1 following house dust mite sensitization in mice. Clin Exp Allergy. (2016) 46:1075–82. 10.1111/cea.12750 27138589

[13] Dolence JJ Kobayashi T Iijima K Krempski J Drake LY Dent AL. Airway exposure initiates peanut allergy by involving the IL-1 pathway and T follicular helper cells in mice. J Allergy Clin Immunol. (2018) 142:1144–58.e8. 10.1016/j.jaci.2017.11.020 29247716 PMC6002896

[14] Liang HE Reinhardt RL Bando JK Sullivan BM Ho IC Locksley RM. Divergent expression patterns of IL-4 and IL-13 define unique functions in allergic immunity. Nat Immunol. (2011) 13:58–66. 10.1038/ni.2182 22138715 PMC3242938

[15] Morita R Schmitt N Bentebibel SE Ranganathan R Bourdery L Zurawski G. Human blood CXCR5(+)CD4(+) T cells are counterparts of T follicular cells and contain specific subsets that differentially support antibody secretion. Immunity. (2011) 34:108–21. 10.1016/j.immuni.2010.12.012 21215658 PMC3046815

[16] Gowthaman U Chen JS Zhang B Flynn WF Lu Y Song W. Identification of a T follicular helper cell subset that drives anaphylactic IgE. Science. (2019) 365:eaaw6433. 10.1126/science.aaw6433 31371561 PMC6901029

[17] Stone KD Prussin C Metcalfe DD IgE, mast cells, basophils, and eosinophils. J Allergy Clin Immunol. (2010) 125:S73–80. 10.1016/j.jaci.2009.11.017 20176269 PMC2847274

[18] Suzuki R Leach S Liu W Ralston E Scheffel J Zhang W. Molecular editing of cellular responses by the high-affinity receptor for IgE. Science. (2014) 343:1021–5. 10.1126/science.1246976 24505132 PMC4188507

[19] Wang J Lin J Bardina L Goldis M Nowak-Wegrzyn A Shreffler WG. Correlation of IgE/IgG4 milk epitopes and affinity of milk-specific IgE antibodies with different phenotypes of clinical milk allergy. J Allergy Clin Immunol. (2010) 125:695–702. 10.1016/j.jaci.2009.12.017 20226304 PMC2841053

[20] Oettgen HC Geha RS. IgE regulation and roles in asthma pathogenesis. J Allergy Clin Immunol. (2001) 107:429–40. 10.1067/mai.2001.113759 11240941

[21] Woltmann G McNulty CA Dewson G Symon FA Wardlaw AJ. Interleukin-13 induces PSGL-1/P-selectin-dependent adhesion of eosinophils, but not neutrophils, to human umbilical vein endothelial cells under flow. Blood. (2000) 95:3146–52. 10.1182/blood.V95.10.3146.010k24_3146_3152 10807781

[22] Johansson MW Annis DS Mosher DF. alpha(M)beta(2) integrin-mediated adhesion and motility of IL-5-stimulated eosinophils on periostin. Am J Respir Cell Mol Biol. (2013) 48:503–10. 10.1165/rcmb.2012-0150OC 23306834 PMC3653603

[23] Bochner BS Klunk DA Sterbinsky SA Coffman RL Schleimer RP. IL-13 selectively induces vascular cell adhesion molecule-1 expression in human endothelial cells. J Immunol. (1995) 154:799–803. 7529288

[24] Li L Xia Y Nguyen A Lai YH Feng L Mosmann TR. Effects of Th2 cytokines on chemokine expression in the lung: IL-13 potently induces eotaxin expression by airway epithelial cells. J Immunol. (1999) 162:2477–87. 10072486

[25] Dubois GR Schweizer RC Versluis C Bruijnzeel-Koomen CA Bruijnzeel PL. Human eosinophils constitutively express a functional interleukin-4 receptor: interleukin-4 -induced priming of chemotactic responses and induction of PI-3 kinase activity. Am J Respir Cell Mol Biol. (1998) 19:691–9. 10.1165/ajrcmb.19.4.3208 9761767

[26] Dubois GR Bruijnzeel-Koomen CA Bruijnzeel PL. IL-4 induces chemotaxis of blood eosinophils from atopic dermatitis patients, but not from normal individuals. J Invest Dermatol. (1994) 102:843–6. 10.1111/1523-1747.ep12382362 8006446

[27] Schleimer RP Sterbinsky SA Kaiser J Bickel CA Klunk DA Tomioka K McIntyre BW. IL-4 induces adherence of human eosinophils and basophils but not neutrophils to endothelium. Association with expression of VCAM-1. J Immunol. (1992) 148:1086–92. 1371130

[28] Grunig G Warnock M Wakil AE Venkayya R Brombacher F Rennick DM. Requirement for IL-13 independently of IL-4 in experimental asthma. Science. (1998) 282:2261–3. 10.1126/science.282.5397.2261 9856950 PMC3897229

[29] Wills-Karp M Luyimbazi J Xu X Schofield B Neben TY Karp CL. Interleukin-13: central mediator of allergic asthma. Science. (1998) 282:2258–61. 10.1126/science.282.5397.2258 9856949

[30] Takayama G Arima K Kanaji T Toda S Tanaka H Shoji S. Periostin: a novel component of subepithelial fibrosis of bronchial asthma downstream of IL-4 and IL-13 signals. J Allergy Clin Immunol. (2006) 118:98–104. 10.1016/j.jaci.2006.02.046 16815144

[31] McBrien CN Menzies-Gow A The biology of eosinophils and their role in asthma. Front Med. (2017) 4:93 10.3389/fmed.2017.00093 PMC5491677 28713812

[32] Shi H Qin S Huang G Chen Y Xiao C Xu H. Infiltration of eosinophils into the asthmatic airways caused by interleukin 5. Am J Respir Cell Mol Biol. (1997) 16:220–4. 10.1165/ajrcmb.16.3.9070605 9070605

[33] Lee JJ Dimina D Macias MP Ochkur SI McGarry MP O'Neill KR. Defining a link with asthma in mice congenitally deficient in eosinophils. Science. (2004) 305:1773–6. 10.1126/science.1099472 15375267

[34] Shen HH Ochkur SI McGarry MP Crosby JR Hines EM Borchers MT. A causative relationship exists between eosinophils and the development of allergic pulmonary pathologies in the mouse. J Immunol. (2003) 170:3296–305. 10.4049/jimmunol.170.6.3296 12626589

[35] Justice JP Borchers MT Crosby JR Hines EM Shen HH Ochkur SI Ablation of eosinophils leads to a reduction of allergen-induced pulmonary pathology. Am J Physiol Lung Cell Mol Physiol. (2003) 284:L169–78. 10.1152/ajplung.00260.2002 12388345

[36] Mattes J Yang M Mahalingam S Kuehr J Webb DC Simson L. Intrinsic defect in T cell production of interleukin (IL)-13 in the absence of both IL-5 and eotaxin precludes the development of eosinophilia and airways hyperreactivity in experimental asthma. J Exp Med. (2002) 195:1433–44. 10.1084/jem.20020009 12045241 PMC2193548

[37] Humbles AA Lloyd CM McMillan SJ Friend DS Xanthou G McKenna EE. A critical role for eosinophils in allergic airways remodeling. Science. (2004) 305:1776–9. 10.1126/science.1100283 15375268

[38] Kay AB Phipps S Robinson DS. A role for eosinophils in airway remodelling in asthma. Trends Immunol. (2004) 25:477–82. 10.1016/j.it.2004.07.006 15324740

[39] Petta I Dejager L Ballegeer M Lievens S Tavernier J De Bosscher K. The interactome of the glucocorticoid receptor and its influence on the actions of glucocorticoids in combatting inflammatory and infectious diseases. Microbiol Mol Biol Rev. (2016) 80:495–522. 10.1128/MMBR.00064-15 27169854 PMC4867367

[40] Klassen C Karabinskaya A Dejager L Vettorazzi S Van Moorleghem J Luhder F. Airway epithelial cells are crucial targets of glucocorticoids in a mouse model of allergic asthma. J Immunol. (2017) 199:48–61. 10.4049/jimmunol.1601691 28515280

[41] Schwiebert LM Stellato C Schleimer RP. The epithelium as a target of glucocorticoid action in the treatment of asthma. Am J Respir Crit Care Med. (1996) 154:S16–9. 10.1164/ajrccm/154.2_Pt_2.S16 8756782

[42] Surjit M Ganti KP Mukherji A Ye T Hua G Metzger D. Widespread negative response elements mediate direct repression by agonist-liganded glucocorticoid receptor. Cell. (2011) 145:224–41. 10.1016/j.cell.2011.03.027 21496643

[43] Lim HW Uhlenhaut NH Rauch A Weiner J Hubner S Hubner N. Genomic redistribution of GR monomers and dimers mediates transcriptional response to exogenous glucocorticoid *in vivo*. Genome Res. (2015) 25:836–44. 10.1101/gr.188581.114 25957148 PMC4448680

[44] Schiller BJ Chodankar R Watson LC Stallcup MR Yamamoto KR. Glucocorticoid receptor binds half sites as a monomer and regulates specific target genes. Genome Biol. (2014) 15:418. 10.1186/s13059-014-0418-y 25085117 PMC4149261

[45] Rhen T Cidlowski JA. Antiinflammatory action of glucocorticoids–new mechanisms for old drugs. N Engl J Med. (2005) 353:1711–23. 10.1056/NEJMra050541 16236742

[46] De Bosscher K Vanden Berghe W Haegeman G. The interplay between the glucocorticoid receptor and nuclear factor-kappaB or activator protein-1: molecular mechanisms for gene repression. Endocr Rev. (2003) 24:488–522. 10.1210/er.2002-0006 12920152

[47] Bakakos A Loukides S Bakakos P Severe eosinophilic asthma. J Clin Med. (2019) 8:1375 10.3390/jcm8091375 PMC6780074 31480806

[48] MacGlashan DW Bochner BS Adelman DC Jardieu PM Togias A McKenzie-White J. Down-regulation of Fc(epsilon)RI expression on human basophils during *in vivo* treatment of atopic patients with anti-IgE antibody. J Immunol. (1997) 158:1438–45. 9013989

[49] Chang TW Shiung YY. Anti-IgE as a mast cell-stabilizing therapeutic agent. J Allergy Clin Immunol. (2006) 117:1203–12. 10.1016/j.jaci.2006.04.005 16750976

[50] Humbert M Busse W Hanania NA Lowe PJ Canvin J Erpenbeck VJ. Omalizumab in asthma: an update on recent developments. J Allergy Clin Immunol Pract. (2014) 2:525–36.e1. 10.1016/j.jaip.2014.03.010 25213045

[51] Ali AK Hartzema AG. Assessing the association between omalizumab and arteriothrombotic events through spontaneous adverse event reporting. J Asthma Allergy. (2012) 5:1–9. 10.2147/JAA.S29811 22690127 PMC3363016

[52] Patterson MF Borish L Kennedy JL. The past, present, and future of monoclonal antibodies to IL-5 and eosinophilic asthma: a review. J Asthma Allergy. (2015) 8:125–34. 10.2147/JAA.S74178 26604804 PMC4639549

[53] Laviolette M Gossage DL Gauvreau G Leigh R Olivenstein R Katial R. Effects of benralizumab on airway eosinophils in asthmatic patients with sputum eosinophilia. J Allergy Clin Immunol. (2013) 132:1086–96.e5. 10.1016/j.jaci.2013.05.020 23866823 PMC4172321

[54] Kolbeck R Kozhich A Koike M Peng L Andersson CK Damschroder MM. MEDI-563, a humanized anti-IL-5 receptor alpha mAb with enhanced antibody-dependent cell-mediated cytotoxicity function. J Allergy Clin Immunol. (2010) 125:1344–53.e2. 10.1016/j.jaci.2010.04.004 20513525

[55] Nair P Pizzichini MM Kjarsgaard M Inman MD Efthimiadis A Pizzichini E. Mepolizumab for prednisone-dependent asthma with sputum eosinophilia. N Engl J Med. (2009) 360:985–93. 10.1056/NEJMoa0805435 19264687

[56] Haldar P Brightling CE Hargadon B Gupta S Monteiro W Sousa A. Mepolizumab and exacerbations of refractory eosinophilic asthma. N Engl J Med. (2009) 360:973–84. 10.1056/NEJMoa0808991 19264686 PMC3992367

[57] Pavord ID Korn S Howarth P Bleecker ER Buhl R Keene ON. Mepolizumab for severe eosinophilic asthma (DREAM): a multicentre, double-blind, placebo-controlled trial. Lancet. (2012) 380:651–9. 10.1016/S0140-6736(12)60988-X 22901886

[58] Ortega HG Liu MC Pavord ID Brusselle GG FitzGerald JM Chetta A Mepolizumab treatment in patients with severe eosinophilic asthma. N Engl J Med. (2014) 371:1198–207. 10.1056/NEJMoa1403290 25199059

[59] Ortega HG Yancey SW Mayer B Gunsoy NB Keene ON Bleecker ER. Severe eosinophilic asthma treated with mepolizumab stratified by baseline eosinophil thresholds: a secondary analysis of the DREAM and MENSA studies. Lancet Respir Med. (2016) 4:549–56. 10.1016/S2213-2600(16)30031-5 27177493

[60] Bel EH Wenzel SE Thompson PJ Prazma CM Keene ON Yancey SW. Oral glucocorticoid-sparing effect of mepolizumab in eosinophilic asthma. N Engl J Med. (2014) 371:1189–97. 10.1056/NEJMoa1403291 25199060

[61] Yancey SW Bradford ES Keene ON. Disease burden and efficacy of mepolizumab in patients with severe asthma and blood eosinophil counts of >/=150-300cells/muL. Respir Med. (2019) 151:139–41. 10.1016/j.rmed.2019.04.008 31047111

[62] Castro M Mathur S Hargreave F Boulet LP Xie F Young J. Res-5- study, reslizumab for poorly controlled, eosinophilic asthma: a randomized, placebo-controlled study. Am J Respir Crit Care Med. (2011) 184:1125–32. 10.1164/rccm.201103-0396OC 21852542

[63] Castro M Wenzel SE Bleecker ER Pizzichini E Kuna P Busse WW. Benralizumab, an anti-interleukin 5 receptor alpha monoclonal antibody, versus placebo for uncontrolled eosinophilic asthma: a phase 2b randomised dose-ranging study. Lancet Respir Med. (2014) 2:879–90. 10.1016/S2213-2600(14)70201-2 25306557

[64] Castro M Zangrilli J Wechsler ME Bateman ED Brusselle GG Bardin P. Reslizumab for inadequately controlled asthma with elevated blood eosinophil counts: results from two multicentre, parallel, double-blind, randomised, placebo-controlled, phase 3 trials. Lancet Respir Med. (2015) 3:355–66. 10.1016/S2213-2600(15)00042-9 25736990

[65] Bjermer L Lemiere C Maspero J Weiss S Zangrilli J Germinaro M. Reslizumab for inadequately controlled asthma with elevated blood eosinophil levels: a randomized phase 3 study. Chest. (2016) 150:789–98. 10.1016/j.chest.2016.03.032 27056586

[66] Bleecker ER FitzGerald JM Chanez P Papi A Weinstein SF Barker P. Efficacy and safety of benralizumab for patients with severe asthma uncontrolled with high-dosage inhaled corticosteroids and long-acting beta2-agonists (SIROCCO): a randomised, multicentre, placebo-controlled phase 3 trial. Lancet. (2016) 388:2115–27. 10.1016/S0140-6736(16)31324-1 27609408

[67] Park HS Lee SH Lee SY Kim MK Lee BJ Werkstrom V. Efficacy and safety of benralizumab for korean patients with severe, uncontrolled eosinophilic asthma. Allergy Asthma Immunol Res. (2019) 11:508–18. 10.4168/aair.2019.11.4.508 31172719 PMC6557768

[68] Ibrahim H O'Sullivan R Casey D Murphy J MacSharry J Plant BJ. The effectiveness of Reslizumab in severe asthma treatment: a real-world experience. Respir Res. (2019) 20:289. 10.1186/s12931-019-1251-3 31861993 PMC6923853

[69] FitzGerald JM Bleecker ER Nair P Korn S Ohta K Lommatzsch M Goldman, investigators Cs. Benralizumab, an anti-interleukin-5 receptor alpha monoclonal antibody, as add-on treatment for patients with severe, uncontrolled, eosinophilic asthma (CALIMA): a randomised, double-blind, placebo-controlled phase 3 trial. Lancet. (2016) 388:2128–41. 10.1016/S0140-6736(16)31322-8 27609406

[70] Nair P Wenzel S Rabe KF Bourdin A Lugogo NL Kuna P. Oral glucocorticoid-sparing effect of benralizumab in severe asthma. N Engl J Med. (2017) 376:2448–58. 10.1056/NEJMoa1703501 28530840

[71] Leckie MJ Ten Brinke A Khan J Diamant Z O'Connor BJ Walls CM. Effects of an interleukin-5 blocking monoclonal antibody on eosinophils, airway hyper-responsiveness, and the late asthmatic response. Lancet. (2000) 356:2144–8. 10.1016/S0140-6736(00)03496-6 11191542

[72] Flood-Page P Swenson C Faiferman I Matthews J Williams M Brannick L. International mepolizumab study, a study to evaluate safety and efficacy of mepolizumab in patients with moderate persistent asthma. Am J Respir Crit Care Med. (2007) 176:1062–71. 10.1164/rccm.200701-085OC 17872493

[73] Kips JC O'Connor BJ Langley SJ Woodcock A Kerstjens HA Postma DS. Effect of SCH55700, a humanized anti-human interleukin-5 antibody, in severe persistent asthma: a pilot study. Am J Respir Crit Care Med. (2003) 167:1655–9. 10.1164/rccm.200206-525OC 12649124

[74] Hanania NA Korenblat P Chapman KR Bateman ED Kopecky P Paggiaro P. Efficacy and safety of lebrikizumab in patients with uncontrolled asthma (LAVOLTA I and LAVOLTA II): replicate, phase 3, randomised, double-blind, placebo-controlled trials. Lancet Respir Med. (2016) 4:781–96. 10.1016/S2213-2600(16)30265-X 27616196

[75] Corren J Lemanske RF Hanania NA Korenblat PE Parsey MV Arron JR Lebrikizumab treatment in adults with asthma. N Engl J Med. (2011) 365:1088–98. 10.1056/NEJMoa1106469 21812663

[76] Scheerens H Arron JR Zheng Y Putnam WS Erickson RW Choy DF. The effects of lebrikizumab in patients with mild asthma following whole lung allergen challenge. Clin Exp Allergy. (2014) 44:38–46. 10.1111/cea.12220 24131304 PMC4204278

[77] Panettieri RA Sjobring U Peterffy A Wessman P Bowen K Piper E. Tralokinumab for severe, uncontrolled asthma (STRATOS 1 and STRATOS 2): two randomised, double-blind, placebo-controlled, phase 3 clinical trials. Lancet Respir Med. (2018) 6:511–25. 10.1016/S2213-2600(18)30184-X 29792288

[78] Busse WW Brusselle GG Korn S Kuna P Magnan A Cohen D. Tralokinumab did not demonstrate oral corticosteroid-sparing effects in severe asthma. Eur Respir J. (2019) 53:1800948. 10.1183/13993003.00948-2018 30442714

[79] Russell RJ Chachi L FitzGerald JM Backer V Olivenstein R Titlestad IL. Effect of tralokinumab, an interleukin-13 neutralising monoclonal antibody, on eosinophilic airway inflammation in uncontrolled moderate-to-severe asthma (MESOS): a multicentre, double-blind, randomised, placebo-controlled phase 2 trial. Lancet Respir Med. (2018) 6:499–510. 10.1016/S2213-2600(18)30201-7 29793857

[80] Piper E Brightling C Niven R Oh C Faggioni R Poon K. A phase II placebo-controlled study of tralokinumab in moderate-to-severe asthma. Eur Respir J. (2013) 41:330–8. 10.1183/09031936.00223411 22743678 PMC3561510

[81] May RD Monk PD Cohen ES Manuel D Dempsey F Davis NH. Preclinical development of CAT-354, an IL-13 neutralizing antibody, for the treatment of severe uncontrolled asthma. Br J Pharmacol. (2012) 166:177–93. 10.1111/j.1476-5381.2011.01659.x 21895629 PMC3415647

[82] De Boever EH Ashman C Cahn AP Locantore NW Overend P Pouliquen IJ. Efficacy and safety of an anti-IL-13 mAb in patients with severe asthma: a randomized trial. J Allergy Clin Immunol. (2014) 133:989–96. 10.1016/j.jaci.2014.01.002 24582316

[83] Gauvreau GM Boulet LP Cockcroft DW Fitzgerald JM Carlsten C Davis BE. Effects of interleukin-13 blockade on allergen-induced airway responses in mild atopic asthma. Am J Respir Crit Care Med. (2011) 183:1007–14. 10.1164/rccm.201008-1210OC 21057005

[84] Kasaian MT Marquette K Fish S DeClercq C Agostinelli R Cook TA. An IL-4/IL-13 dual antagonist reduces lung inflammation, airway hyperresponsiveness, and IgE production in mice. Am J Respir Cell Mol Biol. (2013) 49:37–46. 10.1165/rcmb.2012-0500OC 23449738

[85] Wenzel S Wilbraham D Fuller R Getz EB Longphre M. Effect of an interleukin-4 variant on late phase asthmatic response to allergen challenge in asthmatic patients: results of two phase 2a studies. Lancet. (2007) 370:1422–31. 10.1016/S0140-6736(07)61600-6 17950857

[86] Aerovance Inc Phase 2b Clinical Trial Results Show Aerovance, Inc.'s Aerovant(TM) is Effective in Patients with Eosinophilic Asthma. BioSpace (2010). Available online at: https://www.prnewswire.com/news-releases/phase-2b-clinical-trial-results-show-aerovances-aerovant-is-effective-in-patients-with-eosinophilic-asthma-95847899.html

[87] Corren J Busse W Meltzer EO Mansfield L Bensch G Fahrenholz J. A randomized, controlled, phase 2 study of AMG 317, an IL-4Ralpha antagonist, in patients with asthma. Am J Respir Crit Care Med. (2010) 181:788–96. 10.1164/rccm.200909-1448OC 20056900

[88] Castro M Corren J Pavord ID Maspero J Wenzel S Rabe KF. Dupilumab efficacy and safety in moderate-to-severe uncontrolled asthma. N Engl J Med. (2018) 378:2486–96. 10.1056/NEJMoa1804092 29782217

[89] Wenzel S Ford L Pearlman D Spector S Sher L Skobieranda F. Dupilumab in persistent asthma with elevated eosinophil levels. N Engl J Med. (2013) 368:2455–66. 10.1056/NEJMoa1304048 23688323

[90] Wenzel S Castro M Corren J Maspero J Wang L Zhang B. Dupilumab efficacy and safety in adults with uncontrolled persistent asthma despite use of medium-to-high-dose inhaled corticosteroids plus a long-acting beta2 agonist: a randomised double-blind placebo-controlled pivotal phase 2b dose-ranging trial. Lancet. (2016) 388:31–44. 10.1016/S0140-6736(16)30307-5 27130691

[91] Rabe KF Nair P Brusselle G Maspero JF Castro M Sher L. Efficacy and safety of dupilumab in glucocorticoid-dependent severe asthma. N Engl J Med. (2018) 378:2475–85. 10.1056/NEJMoa1804093 29782224

[92] Beal AM Ramos-Hernandez N Riling CR Nowelsky EA Oliver PM. TGF-beta induces the expression of the adaptor Ndfip1 to silence IL-4 production during iTreg cell differentiation. Nat Immunol. (2011) 13:77–85. 10.1038/ni.2154 22080920 PMC3542978

[93] Dardalhon V Awasthi A Kwon H Galileos G Gao W Sobel RA. IL-4 inhibits TGF-beta-induced Foxp3+ T cells and, together with TGF-beta, generates IL-9+ IL-10+ Foxp3(-) effector T cells. Nat Immunol. (2008) 9:1347–55. 10.1038/ni.1677 18997793 PMC2999006

[94] Hadjur S Bruno L Hertweck A Cobb BS Taylor B Fisher AG. IL4 blockade of inducible regulatory T cell differentiation: the role of Th2 cells, Gata3 and PU.1. Immunol Lett. (2009) 122:37–43. 10.1016/j.imlet.2008.11.001 19046990

[95] Xu L Kitani A Strober W. Molecular mechanisms regulating TGF-beta-induced Foxp3 expression. Mucosal Immunol. (2010) 3:230–8. 10.1038/mi.2010.7 20404810 PMC3673708

[96] Bachert C Mannent L Naclerio RM Mullol J Ferguson BJ Gevaert P. Effect of subcutaneous dupilumab on nasal polyp burden in patients with chronic sinusitis and nasal polyposis: a randomized clinical trial. JAMA. (2016) 315:469–79. 10.1001/jama.2015.19330 26836729

[97] Simpson EL Bieber T Guttman-Yassky E Beck LA Blauvelt A Cork MJ. Two phase 3 trials of dupilumab versus placebo in atopic dermatitis. N Engl J Med. (2016) 375:2335–48. 10.1056/NEJMoa1610020 27690741

[98] Thaci D Simpson EL Beck LA Bieber T Blauvelt A Papp K. Efficacy and safety of dupilumab in adults with moderate-to-severe atopic dermatitis inadequately controlled by topical treatments: a randomised, placebo-controlled, dose-ranging phase 2b trial. Lancet. (2016) 387:40–52. 10.1016/S0140-6736(15)00388-8 26454361

[99] Godar M Deswarte K Vergote K Saunders M de Haard H Hammad H. A bispecific antibody strategy to target multiple type 2 cytokines in asthma. J Allergy Clin Immunol. (2018) 142:1185–93.e4. 10.1016/j.jaci.2018.06.002 29890236 PMC7610797

[100] Ying S O'Connor B Ratoff J Meng Q Mallett K Cousins D. Thymic stromal lymphopoietin expression is increased in asthmatic airways and correlates with expression of Th2-attracting chemokines and disease severity. J Immunol. (2005) 174:8183–90. 10.4049/jimmunol.174.12.8183 15944327

[101] Ying S O'Connor B Ratoff J Meng Q Fang C Cousins D. Expression and cellular provenance of thymic stromal lymphopoietin and chemokines in patients with severe asthma and chronic obstructive pulmonary disease. J Immunol. (2008) 181:2790–8. 10.4049/jimmunol.181.4.2790 18684970

[102] Li Y Wang W Lv Z Li Y Chen Y Huang K. Elevated expression of IL-33 and TSLP in the airways of human asthmatics *in vivo*: a potential biomarker of severe refractory disease. J Immunol. (2018) 200:2253–62. 10.4049/jimmunol.1701455 29453280

[103] Shikotra Choy DF Ohri CM Doran E Butler C Hargadon B. Increased expression of immunoreactive thymic stromal lymphopoietin in patients with severe asthma. J Allergy Clin Immunol. (2012) 129:104–11.e1–9. 10.1016/j.jaci.2011.08.031 21975173

[104] Soumelis V Reche PA Kanzler H Yuan W Edward G Homey B. Human epithelial cells trigger dendritic cell mediated allergic inflammation by producing TSLP. Nat Immunol. (2002) 3:673–80. 10.1038/ni805 12055625

[105] Bleck B Kazeros A Bakal K Garcia-Medina L Adams A Liu M. Coexpression of type 2 immune targets in sputum-derived epithelial and dendritic cells from asthmatic subjects. J Allergy Clin Immunol. (2015) 136:619–27.e5. 10.1016/j.jaci.2014.12.1950 25813919

[106] Kabata H Flamar AL Mahlakoiv T Moriyama S Rodewald HR Ziegler SF. Targeted deletion of the TSLP receptor reveals cellular mechanisms that promote type 2 airway inflammation. Mucosal Immunol. (2020) 13:626–36. 10.1038/s41385-020-0266-x 32066836 PMC7311324

[107] Tang H Cao W Kasturi SP Ravindran R Nakaya HI Kundu K. The T helper type 2 response to cysteine proteases requires dendritic cell-basophil cooperation via ROS-mediated signaling. Nat Immunol. (2010) 11:608–17. 10.1038/ni.1883 20495560 PMC3145206

[108] Nakanishi W Hiraishi Y Yamaguchi S Takamori A Morita H Matsumoto K. TSLP receptor is not essential for house dust mite-induced allergic rhinitis in mice. Biochem Biophys Rep. (2016) 7:119–23. 10.1016/j.bbrep.2016.06.003 28955898 PMC5613305

[109] Kale SL Agrawal K Gaur SN Arora N. Cockroach protease allergen induces allergic airway inflammation via epithelial cell activation. Sci Rep. (2017) 7:42341. 10.1038/srep42341 28198394 PMC5309839

[110] Li DQ Zhang L Pflugfelder SC De Paiva CS Zhang X Zhao G. Short ragweed pollen triggers allergic inflammation through toll-like receptor 4-dependent thymic stromal lymphopoietin/OX40 ligand/OX40 signaling pathways. J Allergy Clin Immunol. (2011) 128:1318–25.e2. 10.1016/j.jaci.2011.06.041 21820713 PMC3229670

[111] Kouzaki H O'Grady SM Lawrence CB Kita H. Proteases induce production of thymic stromal lymphopoietin by airway epithelial cells through protease-activated receptor-2. J Immunol. (2009) 183:1427–34. 10.4049/jimmunol.0900904 19561109 PMC2706924

[112] Hiraishi Y Yamaguchi S Yoshizaki T Nambu A Shimura E Takamori A. Nakae IL-33 S, IL-25 and TSLP contribute to development of fungal-associated protease-induced innate-type airway inflammation. Sci Rep. (2018) 8:18052. 10.1038/s41598-018-36440-x 30575775 PMC6303299

[113] Halim TY Krauss RH Sun AC Takei F. Lung natural helper cells are a critical source of Th2 cell-type cytokines in protease allergen-induced airway inflammation. Immunity. (2012) 36:451–63. 10.1016/j.immuni.2011.12.020 22425247

[114] Al-Shami Spolski R Kelly J Keane-Myers A Leonard WJ. A role for TSLP in the development of inflammation in an asthma model. J Exp Med. (2005) 202:829–39. 10.1084/jem.20050199 16172260 PMC2212950

[115] Zhou Comeau MR De Smedt T Liggitt HD Dahl ME Lewis DB. Thymic stromal lymphopoietin as a key initiator of allergic airway inflammation in mice. Nat Immunol. (2005) 6:1047–53. 10.1038/ni1247 16142237

[116] Zhou Headley MB Aye T Tocker J Comeau MR Ziegler SF. Reversal of thymic stromal lymphopoietin-induced airway inflammation through inhibition of Th2 responses. J Immunol. (2008) 181:6557–62. 10.4049/jimmunol.181.9.6557 18941246 PMC2878483

[117] Omori M Ziegler S. Induction of IL-4 expression in CD4(+) T cells by thymic stromal lymphopoietin. J Immunol. (2007) 178:1396–404. 10.4049/jimmunol.178.3.1396 17237387

[118] Ito T Wang YH Duramad O Hori T Delespesse GJ Watanabe N. TSLP-activated dendritic cells induce an inflammatory T helper type 2 cell response through OX40 ligand. J Exp Med. (2005) 202:1213–23. 10.1084/jem.20051135 16275760 PMC2213234

[119] Wang YH Ito T Wang YH Homey B Watanabe N Martin R. Maintenance and polarization of human TH2 central memory T cells by thymic stromal lymphopoietin-activated dendritic cells. Immunity. (2006) 24:827–38. 10.1016/j.immuni.2006.03.019 16782037

[120] Halim TY Steer CA Matha L Gold MJ Martinez-Gonzalez I McNagny KM. Group 2 innate lymphoid cells are critical for the initiation of adaptive T helper 2 cell-mediated allergic lung inflammation. Immunity. (2014) 40:425–35. 10.1016/j.immuni.2014.01.011 24613091 PMC4210641

[121] Mohapatra Van Dyken SJ Schneider C Nussbaum JC Liang HE Locksley RM. Group 2 innate lymphoid cells utilize the IRF4-IL-9 module to coordinate epithelial cell maintenance of lung homeostasis. Mucosal Immunol. (2016) 9:275–86. 10.1038/mi.2015.59 26129648 PMC4698110

[122] Halim TY Hwang YY Scanlon ST Zaghouani H Garbi N Fallon PG. Group 2 innate lymphoid cells license dendritic cells to potentiate memory TH2 cell responses. Nat Immunol. (2016) 17:57–64. 10.1038/ni.3294 26523868 PMC4685755

[123] Corren J Ziegler SF TSLP: from allergy to cancer. Nat Immunol. (2019) 20:1603–9. 10.1038/s41590-019-0524-9 31745338

[124] Verstraete K Peelman F Braun H Lopez J Van Rompaey D Dansercoer A. Structure and antagonism of the receptor complex mediated by human TSLP in allergy and asthma. Nat Commun. (2017) 8:14937. 10.1038/ncomms14937 28368013 PMC5382266

[125] Gauvreau GM O'Byrne PM Boulet LP Wang Y Cockcroft D Bigler J. Effects of an anti-TSLP antibody on allergen-induced asthmatic responses. N Engl J Med. (2014) 370:2102–10. 10.1056/NEJMoa1402895 24846652

[126] Corren J Parnes JR Wang L Mo M Roseti SL Griffiths JM Tezepelumab in adults with uncontrolled asthma. N Engl J Med. (2017) 377:936–46. 10.1056/NEJMoa1704064 28877011

[127] Corren J Chen S Callan L Gil EG. The effect of tezepelumab on hospitalizations and emergency department visits in patients with severe asthma. Ann Allergy Asthma Immunol. (2020) 125:211–4. 10.1016/j.anai.2020.05.020 32474159

[128] Bousquet J Lockey R Malling HJ. Allergen immunotherapy: therapeutic vaccines for allergic diseases. A WHO position paper. J Allergy Clin Immunol. (1998) 102:558–62. 10.1016/S0091-6749(98)70271-4 9802362

[129] Durham SR Walker SM Varga EM Jacobson MR O'Brien F Noble W. Long-term clinical efficacy of grass-pollen immunotherapy. N Engl J Med. (1999) 341:468–75. 10.1056/NEJM199908123410702 10441602

[130] Walker SM Pajno GB Lima MT Wilson DR Durham SR. Grass pollen immunotherapy for seasonal rhinitis and asthma: a randomized, controlled trial. J Allergy Clin Immunol. (2001) 107:87–93. 10.1067/mai.2001.112027 11149996

[131] Canonica GW Cox L Pawankar R Baena-Cagnani CE Blaiss M Bonini S. Sublingual immunotherapy: world allergy organization position paper 2013 update. World Allergy Organ J. (2014) 7:6. 10.1186/1939-4551-7-6 24679069 PMC3983904

[132] Moller C Dreborg S Ferdousi HA Halken S Host A Jacobsen L. Pollen immunotherapy reduces the development of asthma in children with seasonal rhinoconjunctivitis (the PAT-study). J Allergy Clin Immunol. (2002) 109:251–6. 10.1067/mai.2002.121317 11842293

[133] Grembiale RD Camporota L Naty S Tranfa CM Djukanovic R Marsico SA. Effects of specific immunotherapy in allergic rhinitic individuals with bronchial hyperresponsiveness. Am J Respir Crit Care Med. (2000) 162:2048–52. 10.1164/ajrccm.162.6.9909087 11112112

[134] Eng PA Borer-Reinhold M Heijnen IA Gnehm HP. Twelve-year follow-up after discontinuation of preseasonal grass pollen immunotherapy in childhood. Allergy. (2006) 61:198–201. 10.1111/j.1398-9995.2006.01011.x 16409196

[135] Marogna M Spadolini I Massolo A Canonica GW Passalacqua G. Long-lasting effects of sublingual immunotherapy according to its duration: a 15-year prospective study. J Allergy Clin Immunol. (2010) 126:969–75. 10.1016/j.jaci.2010.08.030 20934206

[136] Novak N Mete N Bussmann C Maintz L Bieber T Akdis M. Early suppression of basophil activation during allergen-specific immunotherapy by histamine receptor 2. J Allergy Clin Immunol. (2012) 130:1153–8.e2. 10.1016/j.jaci.2012.04.039 22698521

[137] Platts-Mills T Vaughan J Squillace S Woodfolk J Sporik R. Sensitisation, asthma, and a modified Th2 response in children exposed to cat allergen: a population-based cross-sectional study. Lancet. (2001) 357:752–6. 10.1016/S0140-6736(00)04168-4 11253969

[138] Francis JN James LK Paraskevopoulos G Wong C Calderon MA Durham SR. Grass pollen immunotherapy: IL-10 induction and suppression of late responses precedes IgG4 inhibitory antibody activity. J Allergy Clin Immunol. (2008) 121:1120–5.e2. 10.1016/j.jaci.2008.01.072 18374405

[139] Scadding GW Shamji MH Jacobson MR Lee DI Wilson D Lima MT Sublingual grass pollen immunotherapy is associated with increases in sublingual Foxp3-expressing cells and elevated allergen-specific immunoglobulin G4, immunoglobulin A and serum inhibitory activity for immunoglobulin E-facilitated allergen binding to B cells. Clin Exp Allergy. (2010) 40:598–606. 10.1111/j.1365-2222.2010.03462.x 20184605

[140] Nouri-Aria KT Wachholz PA Francis JN Jacobson MR Walker SM Wilcock LK. Grass pollen immunotherapy induces mucosal and peripheral IL-10 responses and blocking IgG activity. J Immunol. (2004) 172:3252–9. 10.4049/jimmunol.172.5.3252 14978133

[141] James LK Shamji MH Walker SM Wilson DR Wachholz PA Francis JN. Long-term tolerance after allergen immunotherapy is accompanied by selective persistence of blocking antibodies. J Allergy Clin Immunol. (2011) 127:509–16.e1–5. 10.1016/j.jaci.2010.12.1080 21281875

[142] Suarez-Fueyo Ramos T Galan A Jimeno L Wurtzen PA Marin A de Frutos C. Grass tablet sublingual immunotherapy downregulates the TH2 cytokine response followed by regulatory T-cell generation. J Allergy Clin Immunol. (2014) 133:130–8.e1–2. 10.1016/j.jaci.2013.09.043 24290282

[143] Jeannin P Lecoanet S Delneste Y Gauchat JF Bonnefoy JY. IgE versus IgG4 production can be differentially regulated by IL-10. J Immunol. (1998) 160:3555–61. 9531318

[144] James LK Till SJ. Potential Mechanisms for IgG4 Inhibition of Immediate Hypersensitivity Reactions. Curr Allergy Asthma Rep. (2016) 16:23. 10.1007/s11882-016-0600-2 26892721 PMC4759210

[145] Scadding GW Eifan AO Lao-Araya M Penagos M Poon SY Steveling E. Effect of grass pollen immunotherapy on clinical and local immune response to nasal allergen challenge. Allergy. (2015) 70:689–96. 10.1111/all.12608 25773990 PMC4826905

[146] Radulovic S Jacobson MR Durham SR Nouri-Aria KT. Grass pollen immunotherapy induces Foxp3-expressing CD4+ CD25+ cells in the nasal mucosa. J Allergy Clin Immunol. (2008) 121:1467–72. 10.1016/j.jaci.2008.03.013 18423565

[147] Secrist H Chelen CJ Wen Y Marshall JD Umetsu DT. Allergen immunotherapy decreases interleukin 4 production in CD4+ T cells from allergic individuals. J Exp Med. (1993) 178:2123–30. 10.1084/jem.178.6.2123 7902409 PMC2191292

[148] Wang CM Chang CB Chan MW Wen ZH Wu SF. Dust mite allergen-specific immunotherapy increases IL4 DNA methylation and induces Der p-specific T cell tolerance in children with allergic asthma. Cell Mol Immunol. (2018) 15:963–72. 10.1038/cmi.2017.26 28603280 PMC6207658

[149] Jutel M Akdis M Budak F Aebischer-Casaulta C Wrzyszcz M Blaser K. IL-10 and TGF-beta cooperate in the regulatory T cell response to mucosal allergens in normal immunity and specific immunotherapy. Eur J Immunol. (2003) 33:1205–14. 10.1002/eji.200322919 12731045

[150] Sakurai D Yonekura S Iinuma T Sakurai T Morimoto Y Mita Y. Sublingual immunotherapy for allergic rhinitis: subjective versus objective tools to evaluate its success. Rhinology. (2016) 54:221–30. 10.4193/Rhin15.223 27107025

[151] Bohle B Kinaciyan T Gerstmayr M Radakovics A Jahn-Schmid B Ebner C. Sublingual immunotherapy induces IL-10-producing T regulatory cells, allergen-specific T-cell tolerance, immune deviation. J Allergy Clin Immunol. (2007) 120:707–13. 10.1016/j.jaci.2007.06.013 17681368

[152] Wambre E DeLong JH James EA LaFond RE Robinson D Kwok WW. Differentiation stage determines pathologic and protective allergen-specific CD4+ T-cell outcomes during specific immunotherapy. J Allergy Clin Immunol. (2012) 129:544–51. 10.1016/j.jaci.2011.08.034 21975176 PMC3268867

[153] Mobs C Slotosch C Loffler H Jakob T Hertl M Pfutzner W. Birch pollen immunotherapy leads to differential induction of regulatory T cells and delayed helper T cell immune deviation. J Immunol. (2010) 184:2194–203. 10.4049/jimmunol.0901379 20048125

[154] Boonpiyathad T Satitsuksanoa P Akdis M Akdis CA. Il-10 producing T and B cells in allergy. Semin Immunol. (2019) 44:101326. 10.1016/j.smim.2019.101326 31711770

[155] Coomes SM Kannan Y Pelly VS Entwistle LJ Guidi R Perez-Lloret J. CD4(+) Th2 cells are directly regulated by IL-10 during allergic airway inflammation. Mucosal Immunol. (2017) 10:150–61. 10.1038/mi.2016.47 27166557

[156] de Waal Malefyt R Haanen J Spits H Roncarolo MG te Velde A Figdor C. Interleukin 10 (IL-10) and viral IL-10 strongly reduce antigen-specific human T cell proliferation by diminishing the antigen-presenting capacity of monocytes via downregulation of class II major histocompatibility complex expression. J Exp Med. (1991) 174:915–24. 10.1084/jem.174.4.915 1655948 PMC2118975

[157] Koppelman B Neefjes JJ de Vries JE R de Waal Malefyt, Interleukin-10 down-regulates MHC class II alphabeta peptide complexes at the plasma membrane of monocytes by affecting arrival and recycling. Immunity. (1997) 7:861–71. 10.1016/S1074-7613(00)80404-5 9430231

[158] R.J. van Neerven Wikborg T Lund G Jacobsen B Brinch-Nielsen A Arnved J. Blocking antibodies induced by specific allergy vaccination prevent the activation of CD4+ T cells by inhibiting serum-IgE-facilitated allergen presentation. J Immunol. (1999) 163:2944–52. 10453043

[159] Holm J Willumsen N Wurtzen PA Christensen LH Lund K. Facilitated antigen presentation and its inhibition by blocking IgG antibodies depends on IgE repertoire complexity. J Allergy Clin Immunol. (2011) 127:1029–37. 10.1016/j.jaci.2011.01.062 21377718

[160] James LK Bowen H Calvert RA Dodev TS Shamji MH Beavil AJ. Allergen specificity of IgG(4)-expressing B cells in patients with grass pollen allergy undergoing immunotherapy. J Allergy Clin Immunol. (2012) 130:663–70 e3. 10.1016/j.jaci.2012.04.006 22583928

[161] Taylor Verhagen J Blaser K Akdis M Akdis CA. Mechanisms of immune suppression by interleukin-10 and transforming growth factor-beta: the role of T regulatory cells. Immunology. (2006) 117:433–42. 10.1111/j.1365-2567.2006.02321.x 16556256 PMC1782242

[162] Ansotegui IJ Melioli G Canonica GW Caraballo L Villa E Ebisawa M. IgE allergy diagnostics and other relevant tests in allergy, a World Allergy Organization position paper. World Allergy Organ J. (2020) 13:100080. 10.1016/j.waojou.2019.100080 32128023 PMC7044795

[163] Bernstein DI Epstein T Murphy-Berendts K Liss GM. Surveillance of systemic reactions to subcutaneous immunotherapy injections: year 1 outcomes of the ACAAI and AAAAI collaborative study. Ann Allergy Asthma Immunol. (2010) 104:530–5. 10.1016/j.anai.2010.04.008 20568387 PMC8246419

[164] Epstein TG Liss GM Murphy-Berendts K Bernstein DI. Immediate and delayed-onset systemic reactions after subcutaneous immunotherapy injections: ACAAI/AAAAI surveillance study of subcutaneous immunotherapy: year 2. Ann Allergy Asthma Immunol. (2011) 107:426–31.e1. 10.1016/j.anai.2011.05.020 22018614 PMC8207523

[165] Bernstein DI Wanner M Borish L Liss GM A.A.o.A.A. Immunotherapy Committee, and Immunology, twelve-year survey of fatal reactions to allergen injections and skin testing: 1990-2001. J Allergy Clin Immunol. (2004) 113:1129–36. 10.1016/j.jaci.2004.02.006 15208595

[166] Eifan AO Akkoc T Yildiz A Keles S Ozdemir C Bahceciler NN. Clinical efficacy and immunological mechanisms of sublingual and subcutaneous immunotherapy in asthmatic/rhinitis children sensitized to house dust mite: an open randomized controlled trial. Clin Exp Allergy. (2010) 40:922–32. 10.1111/j.1365-2222.2009.03448.x 20100188

[167] Keles S Karakoc-Aydiner E Ozen A Izgi AG Tevetoglu A Akkoc T. A novel approach in allergen-specific immunotherapy: combination of sublingual and subcutaneous routes. J Allergy Clin Immunol. (2011) 128:808–15.e7. 10.1016/j.jaci.2011.04.033 21641635

[168] Yukselen Kendirli SG Yilmaz M Altintas DU Karakoc GB. Effect of one-year subcutaneous and sublingual immunotherapy on clinical and laboratory parameters in children with rhinitis and asthma: a randomized, placebo-controlled, double-blind, double-dummy study. Int Arch Allergy Immunol. (2012) 157:288–98. 10.1159/000327566 22041501

[169] Quirino T Iemoli E Siciliani E Parmiani S Milazzo F. Sublingual versus injective immunotherapy in grass pollen allergic patients: a double blind (double dummy) study. Clin Exp Allergy. (1996) 26:1253–61. 10.1111/j.1365-2222.1996.tb00522.x 8955574

[170] Mungan D Misirligil Z Gurbuz L. Comparison of the efficacy of subcutaneous and sublingual immunotherapy in mite-sensitive patients with rhinitis and asthma–a placebo controlled study. Ann Allergy Asthma Immunol. (1999) 82:485–90. 10.1016/S1081-1206(10)62726-3 10353581

[171] Khinchi MS Poulsen LK Carat F Andre C Hansen AB Malling HJ. Clinical efficacy of sublingual and subcutaneous birch pollen allergen-specific immunotherapy: a randomized, placebo-controlled, double-blind, double-dummy study. Allergy. (2004) 59:45–53. 10.1046/j.1398-9995.2003.00387.x 14674933

[172] Calderon MA Simons FE Malling HJ Lockey RF Moingeon P Demoly P. Sublingual allergen immunotherapy: mode of action and its relationship with the safety profile. Allergy. (2012) 67:302–11. 10.1111/j.1398-9995.2011.02761.x 22150126

[173] Bachus H Kaur K Papillion AM Marquez-Lago TT Yu Z Ballesteros-Tato A. Impaired tumor-necrosis-factor-alpha-driven dendritic cell activation limits lipopolysaccharide-induced protection from allergic inflammation in infants. Immunity. (2019) 50:225–40.e4. 10.1016/j.immuni.2018.11.012 30635238 PMC6335154

[174] Gereda JE Leung DY Thatayatikom A Streib JE Price MR Klinnert MD. Relation between house-dust endotoxin exposure, type 1 T-cell development, and allergen sensitisation in infants at high risk of asthma. Lancet. (2000) 355:1680–3. 10.1016/S0140-6736(00)02239-X 10905243

[175] Stein MM Hrusch CL Gozdz J Igartua C Pivniouk V Murray SE. Innate immunity and asthma risk in amish and hutterite farm children. N Engl J Med. (2016) 375:411–21. 10.1056/NEJMoa1508749 27518660 PMC5137793

[176] Baldridge JR McGowan P Evans JT Cluff C Mossman S Johnson D. Taking a toll on human disease: toll-like receptor 4 agonists as vaccine adjuvants and monotherapeutic agents. Expert Opin Biol Ther. (2004) 4:1129–38. 10.1517/14712598.4.7.1129 15268679

[177] Chentouh R Fitting C Cavaillon JM. Specific features of human monocytes activation by monophosphoryl lipid A. Sci Rep. (2018) 8:7096. 10.1038/s41598-018-25367-y 29728623 PMC5935727

[178] Drachenberg KJ Wheeler AW Stuebner P Horak F. A well-tolerated grass pollen-specific allergy vaccine containing a novel adjuvant, monophosphoryl lipid A, reduces allergic symptoms after only four preseasonal injections. Allergy. (2001) 56:498–505. 10.1034/j.1398-9995.2001.056006498.x 11421893

[179] Mothes N Heinzkill M Drachenberg KJ Sperr WR Krauth MT Majlesi Y. Allergen-specific immunotherapy with a monophosphoryl lipid A-adjuvanted vaccine: reduced seasonally boosted immunoglobulin E production and inhibition of basophil histamine release by therapy-induced blocking antibodies. Clin Exp Allergy. (2003) 33:1198–208. 10.1046/j.1365-2222.2003.01699.x 12956739

[180] Patel P Salapatek AM. Pollinex Quattro: a novel and well-tolerated, ultra short-course allergy vaccine. Expert Rev Vaccines. (2006) 5:617–29. 10.1586/14760584.5.5.617 17181436

[181] Zielen S Gabrielpillai J Herrmann E Schulze J Schubert R Rosewich M. Long-term effect of monophosphoryl lipid A adjuvanted specific immunotherapy in patients with grass pollen allergy. Immunotherapy. (2018) 10:529–36. 10.2217/imt-2018-0004 29562801

[182] DuBuske LM Frew AJ Horak F Keith PK Corrigan CJ Aberer W. Ultrashort-specific immunotherapy successfully treats seasonal allergic rhinoconjunctivitis to grass pollen. Allergy Asthma Proc. (2011) 32:239–47. 10.2500/aap.2011.32.3453 21535913

[183] Rosewich M Girod K Zielen S Schubert R Schulze J. Induction of bronchial tolerance after 1 cycle of monophosphoryl-A-adjuvanted specific immunotherapy in children with grass pollen allergies. Allergy Asthma Immunol Res. (2016) 8:257–63. 10.4168/aair.2016.8.3.257 26922936 PMC4773214

[184] Pfaar O Barth C Jaschke C Hormann K Klimek L. Sublingual allergen-specific immunotherapy adjuvanted with monophosphoryl lipid A: a phase I/IIa study. Int Arch Allergy Immunol. (2011) 154:336–44. 10.1159/000321826 20975285

[185] Patel P Holdich T Fischer von Weikersthal-Drachenberg KJ Huber B. Efficacy of a short course of specific immunotherapy in patients with allergic rhinoconjunctivitis to ragweed pollen. J Allergy Clin Immunol. (2014) 133:121–9.e1–2. 10.1016/j.jaci.2013.05.032 23870670

[186] Worm M Higenbottam T Pfaar O Mosges R Aberer W Gunawardena K. Randomized controlled trials define shape of dose response for Pollinex Quattro Birch allergoid immunotherapy. Allergy. (2018) 73:1812–22. 10.1111/all.13478 29779247 PMC6175210

[187] Creticos PS Schroeder JT Hamilton RG Balcer-Whaley SL Khattignavong AP Lindblad R. Immune tolerance network, immunotherapy with a ragweed-toll-like receptor 9 agonist vaccine for allergic rhinitis. N Engl J Med. (2006) 355:1445–55. 10.1056/NEJMoa052916 17021320

[188] Senti G Johansen P Haug S Bull C Gottschaller C Muller P. Use of A-type CpG oligodeoxynucleotides as an adjuvant in allergen-specific immunotherapy in humans: a phase I/IIa clinical trial. Clin Exp Allergy. (2009) 39:562–70. 10.1111/j.1365-2222.2008.03191.x 19226280

[189] Klimek L Willers J Hammann-Haenni A Pfaar O Stocker H Mueller P. Assessment of clinical efficacy of CYT003-QbG10 in patients with allergic rhinoconjunctivitis: a phase IIb study. Clin Exp Allergy. (2011) 41:1305–12. 10.1111/j.1365-2222.2011.03783.x 21672053

[190] Tsitoura D Ambery C Price M Powley W Garthside S Biggadike K. Early clinical evaluation of the intranasal TLR7 agonist GSK2245035: use of translational biomarkers to guide dosing and confirm target engagement. Clin Pharmacol Ther. (2015) 98:369–80. 10.1002/cpt.157 26044169

[191] Ellis AK Tsitoura DC Quint D Powley W Lee LA. Safety and pharmacodynamics of intranasal GSK2245035, a TLR7 agonist for allergic rhinitis: a randomized trial. Clin Exp Allergy. (2017) 47:1193–203. 10.1111/cea.12974 28681506

[192] Greiff L Cervin A Ahlstrom-Emanuelsson C Almqvist G Andersson M Dolata J. Repeated intranasal TLR7 stimulation reduces allergen responsiveness in allergic rhinitis. Respir Res. (2012) 13:53. 10.1186/1465-9921-13-53 22726593 PMC3487914

[193] Greiff L Ahlstrom-Emanuelsson C Alenas M Almqvist G Andersson M Cervin A. Biological effects and clinical efficacy of a topical Toll-like receptor 7 agonist in seasonal allergic rhinitis: a parallel group controlled phase IIa study. Inflamm Res. (2015) 64:903–15. 10.1007/s00011-015-0873-2 26342289

[194] Senti G Freiburghaus AU Larenas-Linnemann D Hoffmann HJ Patterson AM Klimek L. Intralymphatic immunotherapy: update and unmet needs. Int Arch Allergy Immunol. (2019) 178:141–9. 10.1159/000493647 30391954

[195] Plantinga M Guilliams M Vanheerswynghels M Deswarte K Branco-Madeira F Toussaint W. Conventional and monocyte-derived CD11b(+) dendritic cells initiate and maintain T helper 2 cell-mediated immunity to house dust mite allergen. Immunity. (2013) 38:322–35. 10.1016/j.immuni.2012.10.016 23352232

[196] Coquet JM Schuijs MJ Smyth MJ Deswarte K Beyaert R Braun H. Interleukin-21-producing CD4(+) T cells promote type 2 immunity to house dust mites. Immunity. (2015) 43:318–30. 10.1016/j.immuni.2015.07.015 26287681

[197] Johnston RJ Poholek AC DiToro D Yusuf I Eto D Barnett B. Bcl6 and Blimp-1 are reciprocal and antagonistic regulators of T follicular helper cell differentiation. Science. (2009) 325:1006–10. 10.1126/science.1175870 19608860 PMC2766560

[198] Nurieva RI Chung Y Martinez GJ Yang XO Tanaka S Matskevitch TD. Bcl6 mediates the development of T follicular helper cells. Science. (2009) 325:1001–5. 10.1126/science.1176676 19628815 PMC2857334

[199] Yu D Rao S Tsai LM Lee SK He Y Sutcliffe EL. The transcriptional repressor Bcl-6 directs T follicular helper cell lineage commitment. Immunity. (2009) 31:457–68. 10.1016/j.immuni.2009.07.002 19631565

[200] Yusuf I Kageyama R Monticelli L Johnston RJ Ditoro D Hansen K. Germinal center T follicular helper cell IL-4 production is dependent on signaling lymphocytic activation molecule receptor (CD150). J Immunol. (2010) 185:190–202. 10.4049/jimmunol.0903505 20525889 PMC2913439

[201] Weinstein JS Laidlaw BJ Lu Y Wang JK Schulz VP Li N. STAT4 and T-bet control follicular helper T cell development in viral infections. J Exp Med. (2018) 215:337–55. 10.1084/jem.20170457 29212666 PMC5748849

[202] Luthje K Kallies A Shimohakamada Y Belz GT Light A Tarlinton DM. The development and fate of follicular helper T cells defined by an IL-21 reporter mouse. Nat Immunol. (2012) 13:491–8. 10.1038/ni.2261 22466669

[203] Wojciechowski W Harris DP Sprague F Mousseau B Makris M Kusser K. Cytokine-producing effector B cells regulate type 2 immunity to *H*. polygyrus. Immunity. (2009) 30:421–33. 10.1016/j.immuni.2009.01.006 19249230 PMC2745290

[204] Hsu HC Yang P Wang J Wu Q Myers R Chen J. Interleukin 17-producing T helper cells and interleukin 17 orchestrate autoreactive germinal center development in autoimmune BXD2 mice. Nat Immunol. (2008) 9:166–75. 10.1038/ni1552 18157131

[205] Leon B Ballesteros-Tato A Browning JL Dunn R Randall TD Lund FE. Regulation of T(H)2 development by CXCR5+ dendritic cells and lymphotoxin-expressing B cells. Nat Immunol. (2012) 13:681–90. 10.1038/ni.2309 22634865 PMC3548431

[206] Glatman Zaretsky A Taylor JJ King IL Marshall FA Mohrs M Pearce EJ. T follicular helper cells differentiate from Th2 cells in response to helminth antigens. J Exp Med. (2009) 206:991–9. 10.1084/jem.20090303 19380637 PMC2715032

[207] Weinstein JS Herman EI Lainez B Licona-Limon P Esplugues E Flavell R. TFH cells progressively differentiate to regulate the germinal center response. Nat Immunol. (2016) 17:1197–205. 10.1038/ni.3554 27573866 PMC5030190

[208] Reinhardt RL Liang HE Locksley RM. Cytokine-secreting follicular T cells shape the antibody repertoire. Nat Immunol. (2009) 10:385–93. 10.1038/ni.1715 19252490 PMC2714053

[209] Johnston RJ Choi YS Diamond JA Yang JA Crotty S. STAT5 is a potent negative regulator of TFH cell differentiation. J Experi Med. (2012) 209:243–50. 10.1084/jem.20111174 22271576 PMC3281266

[210] Nurieva RI Podd A Chen Y Alekseev AM Yu M Qi X. STAT5 protein negatively regulates T follicular helper (Tfh) cell generation and function. J Biol Chem. (2012) 287:11234–9. 10.1074/jbc.M111.324046 22318729 PMC3322890

[211] Leon B Bradley JE Lund FE Randall TD Ballesteros-Tato A. FoxP3+ regulatory T cells promote influenza-specific Tfh responses by controlling IL-2 availability. Nat Commun. (2014) 5:3495. 10.1038/ncomms4495 24633065 PMC4013682

[212] Ballesteros-Tato Leon B Graf BA Moquin A Adams PS Lund FE. Interleukin-2 inhibits germinal center formation by limiting T follicular helper cell differentiation. Immunity. (2012) 36:847–56. 10.1016/j.immuni.2012.02.012 22464171 PMC3361521

[213] Botta D Fuller MJ Marquez-Lago TT Bachus H Bradley JE Weinmann AS. Dynamic regulation of T follicular regulatory cell responses by interleukin 2 during influenza infection. Nat Immunol. (2017) 18:1249–60. 10.1038/ni.3837 28892471 PMC5679073

[214] Papillion Powell MD Chisolm DA Bachus H Fuller MJ Weinmann AS. Inhibition of IL-2 responsiveness by IL-6 is required for the generation of GC-TFH cells. Sci Immunol. (2019) 4. 10.1126/sciimmunol.aaw7636 31519812 PMC6820141

[215] Locci M Wu JE Arumemi F Mikulski Z Dahlberg C Miller AT. Activin A programs the differentiation of human TFH cells. Nat Immunol. (2016) 17:976–84. 10.1038/ni.3494 27376469 PMC4955732

[216] Oestreich KJ Read KA Gilbertson SE Hough KP McDonald PW Krishnamoorthy V. Bcl-6 directly represses the gene program of the glycolysis pathway. Nat Immunol. (2014) 15:957–64. 10.1038/ni.2985 25194422 PMC4226759

[217] He Zhang X Wei Y Sun X Chen Y Deng J. Low-dose interleukin-2 treatment selectively modulates CD4(+) T cell subsets in patients with systemic lupus erythematosus. Nat Med. (2016) 22:991–3. 10.1038/nm.4148 27500725

[218] Ballesteros-Tato Papillion A. Mechanisms of action of low-dose IL-2 restoration therapies in SLE. Curr Opin Immunol. (2019) 61:39–45. 10.1016/j.coi.2019.07.003 31450016

[219] Saadoun D Rosenzwajg M Joly F Six A Carrat F Thibault V. Regulatory T-cell responses to low-dose interleukin-2 in HCV-induced vasculitis. N Engl J Med. (2011) 365:2067–77. 10.1056/NEJMoa1105143 22129253

[220] Koreth J Matsuoka K Kim HT McDonough SM Bindra B Alyea EP. Interleukin-2 and regulatory T cells in graft-versus-host disease. N Engl J Med. (2011) 365:2055–66. 10.1056/NEJMoa1108188 22129252 PMC3727432

